# Mito-TIPTP Increases Mitochondrial Function by Repressing the Rubicon-p22phox Interaction in Colitis-Induced Mice

**DOI:** 10.3390/antiox10121954

**Published:** 2021-12-06

**Authors:** Jae-Sung Kim, Ye-Ram Kim, Sein Jang, Sang Geon Wang, Euni Cho, Seok-Jun Mun, Hye-In Jeon, Hyo-Keun Kim, Sun-Joon Min, Chul-Su Yang

**Affiliations:** 1 Department of Bionano Technology, Hanyang University, Seoul 04673, Korea; sung901017@hanyang.ac.kr (J.-S.K.); chocokr@hanyang.ac.kr (Y.-R.K.); eunicho@hanyang.ac.kr (E.C.); moon07101@hanyang.ac.kr (S.-J.M.); 2 Institute of Natural Science & Technology, Hanyang University, Ansan 15588, Korea; 3 Center for Bionano Intelligence Education and Research, Ansan 15588, Korea; tpdls26@hanyang.ac.kr (S.J.); dhkdtkdrjs@hanyang.ac.kr (S.G.W.); jhi1007@hanyang.ac.kr (H.-I.J.); gyrmsdl103@hanyang.ac.kr (H.-K.K.); 4 Department of Molecular and Life Science, Hanyang University, Ansan 15588, Korea; 5 Department of Applied Chemistry, Hanyang University, Ansan 15588, Korea; 6 Department of Chemical & Molecular Engineering, Hanyang University, Ansan 15588, Korea

**Keywords:** Rubicon, p22phox, mitochondria, reactive oxygen species, colitis

## Abstract

The run/cysteine-rich-domain-containing Beclin1-interacting autophagy protein (Rubicon) is essential for the regulation of nicotinamide adenine dinucleotide phosphate (NADPH) oxidase by interacting with p22phox to trigger the production of reactive oxygen species (ROS) in immune cells. In a previous study, we demonstrated that the interaction of Rubicon with p22phox increases cellular ROS levels. The correlation between Rubicon and mitochondrial ROS (mtROS) is poorly understood. Here, we report that Rubicon interacts with p22phox in the outer mitochondrial membrane in macrophages and patients with human ulcerative colitis. Upon lipopolysaccharide (LPS) activation, the binding of Rubicon to p22phox was elevated, and increased not only cellular ROS levels but also mtROS, with an impairment of mitochondrial complex III and mitochondrial biogenesis in macrophages. Furthermore, increased Rubicon decreases mitochondrial metabolic flux in macrophages. Mito-TIPTP, which is a p22phox inhibitor containing a mitochondrial translocation signal, enhances mitochondrial function by inhibiting the association between Rubicon and p22phox in LPS-primed bone-marrow-derived macrophages (BMDMs) treated with adenosine triphosphate (ATP) or dextran sulfate sodium (DSS). Remarkably, Mito-TIPTP exhibited a therapeutic effect by decreasing mtROS in DSS-induced acute or chronic colitis mouse models. Thus, our findings suggest that Mito-TIPTP is a potential therapeutic agent for colitis by inhibiting the interaction between Rubicon and p22phox to recover mitochondrial function.

## 1. Introduction

Oxygen-derived free radicals, such as superoxide and hydroxide, are known as reactive oxygen species (ROS) and are small, highly reactive molecules. ROS plays an important role in both physiological and pathological cellular responses, depending on ROS concentration. ROS production is tightly regulated to specific subcellular sites and involves complex cellular signaling and negative feedback mechanisms. However, under specific conditions, excessive ROS production results in oxidative stress, causing damage to host intracellular systems, and consequently, various diseases, such as sepsis, inflammatory bowel diseases (IBDs), atherosclerosis, heart failure, cancer, aging, and neurodegeneration [1,2,3].

Mitochondria are membrane-bound organelles that are essential for maintaining energy through oxidative phosphorylation and other metabolic functions. They are also the primary source of ROS. Mitochondrial ROS (mtROS) regulates inflammatory signaling; however, ROS overproduction can lead to oxidative damage of cell components, such as proteins, membranes, and DNA, resulting in mitochondrial dysfunction. Mitochondria dysfunction is the underlying basis for the pathogenesis of various diseases, including metabolic disorders, age-related diseases, and chronic inflammatory states. Studies have demonstrated that perturbed intestinal function can occur in response to mitochondrial dysregulation [4,5,6]. In particular, an upregulation of mtROS levels was observed in IBD patients [7]. Given the importance of mitochondrial function to the intestinal immune system, targeting the regulation of mtROS may represent a promising therapeutic strategy to decrease the pathogenesis of colitis [8].

IBDs are idiopathic, relapsing, and chronic inflammatory disorders of the intestinal tract and colon region. They occur at any age and are accompanied by symptoms such as diarrhea, bloody stools, abdominal pain, and weight loss. Although the exact pathophysiology of IBDs is not fully understood, it is known that the disorder is triggered by multiple factors, such as genetic background, environmental factors, diet, lifestyle, and smoking, gut microbiota, and immune dysregulation [9]. IBDs may be classified into two main types: Crohn’s disease, which can affect a discontinuous patch of the gastrointestinal tract from the mouth to the anus, and ulcerative colitis, which affects only the large intestine [10]. IBDs are lifelong diseases, and treatment consists of long-term therapies that create a tremendous financial burden. Current IBD therapies include anti-inflammatory reagents; 5-aminosalicylates and mesalazines, immunosuppressants; corticosteroid, biological and biosimilar medicines; antibodies, anti-cytokines, anti-adhesion molecules, and fecal microbiota transplant. These therapies may control the severity of IBDs, but there is no overall effective treatment, and a significant percentage of patients fail to respond or lose response to therapy [11,12]. Therefore, new IBD therapeutics are needed, with higher efficacy, precision, and safety for long-term therapy.

We previously discovered that the Run/cysteine-rich-domain-containing Beclin1-interacting autophagy protein (Rubicon) is an essential positive regulator of the NADPH oxidase complex through the interaction with p22phox. This factor facilitates its stabilization and phagosomal trafficking to induce a burst of ROS upon microbial infection or inflammatory stimulation [13]. Furthermore, we recently reported that an N-terminal eight amino acid (N8) peptide derived from p22phox and mimetic of N8, known as 2-(tetrahydroindazolyl)phenoxy-*N*-(thiadiazolyl) propenamide 2 (TIPTP), exhibits potent anti-inflammatory effects by interrupting the interaction of Rubicon and p22phox. These effects protected mice from acute polymicrobial sepsis induced by a cecal ligation procedure (CLP) and chronic rheumatoid arthritis (RA) [14,15]. Thus, these compounds may represent an essential resource for the development of therapeutics against intestinal inflammation.

In the present study, we show that the interaction between Rubicon and p22phox contributes not only to burst production of cytosolic ROS, but also mtROS. We found that Rubicon and p22phox translocate and strongly interact within the outer mitochondrial membrane during inflammatory conditions by cellular fractionation and immunoprecipitation (IP) in bone-marrow-derived macrophages (BMDMs). Additionally, Rubicon contributes to mitochondrial biogenesis and activity by upregulating the expression of related genes in LPS-treated BMDMs. Furthermore, we developed Mito-TIPTP, a p22phox N8 mimetic compound conjugated with triphenylphosphonium (TPP) cation, which traverses phospholipid bilayers, translocates within mitochondria, and targets mitochondria. Mito-TIPTP showed the resolution of excessive mtROS in BMDMs and DSS-induced colitis mice. Furthermore, treatment with Mito-TIPTP protected DSS-induced colitis mice by reducing mtROS generation and restoring mitochondrial metabolism. Thus, the selective inhibition of p22phox-Rubicon using Mito-TIPTP within the mitochondria is a potential treatment strategy for intestinal inflammation.

## 2. Materials and Methods

### 2.1. Mice and Cell Culture

Wild-type (WT) C57BL/6 mice were purchased from Samtako Bio Korea (Gyeonggi-do, Korea). Primary BMDMs were isolated from C57BL/6 mice and cultured in DMEM for 3–5 d in the presence of M-CSF (R&D Systems, 416-ML), as previously described [16]. BMDMs of p22phox^−/−^ in C57BL/6 mice were a generous gift from Dr. Chul-Ho Lee (Laboratory Animal Center, Korea Research Institute of Bioscience and Biotechnology, Daejeon, Korea). The mouse macrophage cell lines RAW264.7 (ATCC TIB-71; American Type Culture Collection) and HEK293T (ATCC-11268) cells were maintained in DMEM (Invitrogen, Waltham, MA, USA) containing 10% FBS (Invitrogen, Waltham, MA, USA), sodium pyruvate, nonessential amino acids, penicillin G (100 IU/mL), and streptomycin (100 μg/mL) (Gibco, New York, NY, USA). Human monocytic cell line THP-1 (ATCC TIB-202) cells were grown in RPMI 1640/glutamax (Gibco, New York, NY, USA) supplemented with 10% FBS (Invitrogen, Waltham, MA, USA).

### 2.2. Reagents and Antibodies

Lipopolysacchride (LPS) (*Escherichia coli* O111:B4, L2630) and adenosine triphosphate (ATP) were purchased from Sigma-Aldrich (St. Louis, MO, USA). Dextran sulfate sodium (DSS, 0216011010) salt (36,000–50,000 MW) was purchased from MP Biomedicals (Santa Ana, CA, USA). Specific antibodies against Rubicon (ab92388), ABCB10 (ab231535), NDUFA9 (ab14713), NDUFA8 (ab184952), UQCRQ (ab241983), ATP5A (ab14748), and GST (ab138491) were purchased from Abcam (Cambridge, UK). Abs specific for VDAC1/Porin (B-6), FACL4 (N-18), Lamin B1 (B-10), Tubulin (5F131), p22phox (FL-195), SDHA (F-2), UQCRC2 (G-10), PGC-1α (D-5), PGC-1β (E-9), NRF1 (H-4), NRF2 (G-2), Tfam (F-6), and Actin (I-19) were purchased from Santa Cruz Biotechnology (Dallas, TX, USA). The antibody to calreticulin (D3E6, 12238), COX IV (4D11-B3-E8, 11967) was purchased from Cell Signaling Technology (Danvers, MA, USA).

### 2.3. Enzyme-Linked Immunosorbent Assay (ELISA)

Colon lysates were analyzed for cytokine content using the BD OptEIA ELISA set (BD Pharmingen, San Diego, CA, USA) for the detection of TNF-α, IL-6, IL-1β, and IL-18. The plates were coated with capture antibody for O.N at 4 °C. After coating, samples were incubated for 2 h at RT, followed by treating the detection antibody in plates for 1 h at RT. After treating detection antibody, streptavidin-HRP was incubated in plates for 30 min at RT, and TMB substrate solution is added to each well for 30 min at RT. Next, stop solution is added to each well and the plates were measured at 450 nm using MMR SPARK^®^ Microplate Reader (Männedorf, Switzerland). All assays were performed as recommended by the manufacturer.

### 2.4. Glutathione S-Transferase (GST) Pulldown, Immunoblot, and Immunoprecipitation Analysis

GST pulldown, immunoprecipitation, and immunoblot assays were performed as previously described [13,16]. For GST pulldown, 293T cells were harvested and lysed in NP-40 buffer supplemented with a complete protease inhibitor cocktail (Roche, Basal, Switzerland). After centrifugation, the supernatants were precleared with protein A/G beads at 4 °C for 2 h. Pre-cleared lysates were mixed with a 50% slurry of glutathione-conjugated Sepharose beads (Amersham Biosciences, Amersham, UK), and the binding reaction was incubated for 4 h at 4 °C. Precipitates were washed extensively with lysis buffer. Proteins bound to glutathione beads were eluted with Sodium Dodecyl Sulfate (SDS) loading buffer by boiling for 5 min.

For immunoprecipitation, 293T cells and BMDMs were harvested and then lysed in NP-40 buffer supplemented with a complete protease inhibitor cocktail (Roche, Basal, Switzerland). After pre-clearing with protein A/G agarose beads for 1 h at 4 °C, whole-cell lysates were used for immunoprecipitation with the indicated antibodies. Generally, 1–4 μg of commercial antibody was added to 1 mL of cell lysates and incubated at 4 °C for 8 to 12 h. After the addition of proteins A/G agarose beads for 6 h, immunoprecipitates were extensively washed with lysis buffer and eluted with SDS loading buffer by boiling for 5 min.

For immunoblotting (IB), polypeptides were resolved by SDS-polyacrylamide gel electrophoresis and transferred to a PVDF membrane (Bio-Rad, Hercules, CA, USA). Immuno detection was achieved with specific antibodies. Antibody binding was visualized by chemiluminescence (ECL; Millipore, Burlington, MA, USA) and detected by a Vilber chemiluminescence analyzer (Fusion SL 3; Vilber Lourmat, Collégien, France).

### 2.5. Tissue Distribution of Rubicon 

Various tissues (heart, brain, kidney, spleen, liver, lung, muscle, thymus, intestine and lymph nodes) were separated from C57BL/6 female mice, aged 8 weeks and homogenized. Rubicon expression was assessed by immunoblotting (IB) and whole-cell lysates (WCL) were used for the IB with αActin. Representative images from three independent experiments are shown.

### 2.6. Subcellular Fractionation

Cytosol and mitochondria were isolated from cells using a Mitochondria Fractionation Kit (Active Motif, Carlsbad, CA, USA, 40015) or as described previously [17]. Cytosol, microsomes (endoplasmic reticulum, ER), mitochondria-associated membrane (MAM) fraction and pure mitochondria were isolated from cells using an Endoplasmic Reticulum Isolation Kit (Sigma, St. Louis, MO, USA, ER0100) or as described previously [18,19,20]. Subcellular fractionated proteins were lysed in buffer containing 2% SDS and boiled with 2× reducing sample buffer for SDS-PAGE.

### 2.7. Fractionation of Mitochondria

The isolated mitochondria were resuspended in alkali extraction reagent (0.1 M Na_2_CO_3_, 0.02 M NaHCO3 (pH 10.5)) and sonicated. The suspension was then centrifuged at 1000× *g* for 10 min at 4 °C and the supernatant was collected as mitochondrial soluble fraction. The pellet, an alkali-insoluble fraction, was collected as the mitochondrial membrane fraction. To separate mitochondrial outer-membrane and mitoplast (inner-membrane plus matrix), isolated mitochondria were resuspended in 0.15 mg/mL digitonin and centrifuged at 12,000× *g* for 15 min at 4 °C. The resulting supernatant was collected as an outer-membrane fraction, and the pellet was recovered as a mitoplast [21,22].

### 2.8. Flow Cytometric Measurement of ROS Production

Intracellular ROS levels were measured by flow cytometry from cells cultured in serum-free medium and loaded with the redox-sensitive dye 2 μM dihydroethidium (DHE for O_2_^−^; Calbiochem, San Diego, CA, USA) or 1 μM MitoSox Red (for mitoROS; Calbiochem, San Diego, CA, USA) 13. The 1 × 10^5^ BMDMs were thoroughly and quickly washed with pulse spin and immediately acquired for analyses in FACSCalibur (BD Biosciences, San Jose, CA, USA). The data were plotted using CellQuest software (BD Biosciences, San Jose, CA, USA).

### 2.9. Metabolic Assays

Lactate levels in the medium were determined using a Lactate Assay Kit (Biovision Milpitas, CA, USA), according to the manufacturer’s instructions. In brief, the cells were stimulated with LPS for the indicated periods and the supernatants were then collected and stored at −80 °C to inactivate lactate dehydrogenase. The reaction mix was added to the samples, which were then analyzed on a microplate reader (OD_570_ nm). The NAD^+^ /NADH ratio was measured from whole-cell lysates using a NAD^+^/NADH Quantification Colorimetric Kit (BioVision, Milpitas, CA, USA), according to the manufacturer’s instructions. For real-time analysis of the extracellular acidification rate (ECAR) and the oxygen consumption rate (OCR), BMDMs were analyzed using an XF-24 Extracellular Flux Analyzer (Seahorse Bioscience, North Billerica, MA, USA). In brief, BMDMs were plated in XF-24 cell culture microplates (2 × 10^5^ cells/well in 200 μL) and then stimulated with LPS for the indicated time periods. At the indicated timepoints, the medium was removed, and the cells were washed and analyzed in XF Running Buffer (unbuffered RPMI, 10 mM glucose, 10% FCS, 100 U/mL penicillin/streptomycin, 2 mM l-glutamine, and 20 ng/mL of GM-CSF) according to the manufacturer’s instructions to analyze real-time values of OCR and ECAR. Where indicated, ECAR and/or OCR were analyzed in response to 2 μg/mL oligomycin, 1 μM carbonyl cyanide-p-trifluoromethoxy–pheny lhydrazone (FCCP), 1 μM Actinomycin-A, 2 μM rotenone, 10 mM glucose, and 50 mM 2-deoxy-d-glucose (2-DG) (all from Sigma-Aldrich).

### 2.10. Confocal Fluorescence Microscopy

Immunofluorescence analysis was performed as previously described [13,16]. The 1 × 10^5^ BMDMs were fixed on coverslips with 4% (*w*/*v*) paraformaldehyde in PBS and then permeabilized for 10 min using 0.25% (*v*/*v*) Triton X-100 in PBS at 25 °C. Rubicon or p22phox was detected using a 1/100 dilution of the primary Ab for 1 h at 25 °C. After washing, the appropriate fluorescently labeled secondary Abs were incubated for 1 h at 25 °C. Slides were examined using laser-scanning confocal microscopy (model LSM 800; Zeiss, Jena, Germany).

### 2.11. Flow Cytometry

Flow cytometry data were acquired on a FACSCanto (BD Biosciences, San Diego, CA, USA) and analyzed with FlowJo software (Tree Star, Ashland, OR, USA). To determine expression of cell surface proteins, mAb were incubated at 4 °C for 20–30 min and 5 × 10^6^ BMDMs were fixed using Cytofix/Cytoperm Solution (BD Biosciences, San Jose, CA) and, in some instances followed by mAb incubation to detect intracellular proteins. The following mAb clones were used: F4/80 (BM8, eBioscience, San Diego, CA, USA), Ly-6G (1A8-Ly6g, eBioscience, San Diego, CA, USA), NK1.1 (PK136, eBioscience, San Diego, CA, USA), CD3/CD4 (M2AB, 7E14, eBioscience, San Diego, CA, USA), CD3/CD8 (M2AB, 17D8, eBioscience, San Diego, CA, USA) and CD19/CD220 (771404, RA3–6B2, eBioscience, San Diego, CA, USA).

### 2.12. Study Population of Human Normal and UC Patients

Two types of samples were included in this study. All participants, as both patients and human normal, provided informed consent, and the patients were enrolled based on their diagnosis prior to chemotherapy treatment. We collected colon biopsy from 20 individuals: 10 human normal (median age 46.6 ± 12.7 y; male 43.2%) and 10 UC patients (median age 49.0 ± 18.4 y; male 43.8%). All participants provided written informed consent regarding the use of their clinical data for research purposes. 

### 2.13. Adenovirus Construction

Both an adenovirus encoding a full-length mouse Rubicon and a Rubicon-specific shRNA adenovirus were constructed, as previously described [15]. The recombinant viruses were amplified in AD-293 cells, purified and concentrated by BD Adeno-X^TM^ purification kit (BD Biosciences, San Jose, CA, USA). The typical titers were in the range of 10^12^–10^13^ plaque-forming units (pfu)/mL as determined via plaque assay using 1.25% SeaPlaque GTG agarose (BioWhittaker, Walkersville, MD, USA) overlay. A sterile carrier solution (PBS) was used for control injections and dilution of the viruses. 

### 2.14. Injection of Recombinant Adenoviruses for Depletion of Rubicon in Mice

Recombinant adenoviruses (Ad-vector or Ad-Rubicon) were intravenously injected via the tail vein at a dose of ~1 × 10^12^ pfu/mouse twice. At 2 days post-transduction, mice were challenged with DSS. 

### 2.15. Mouse Model of Colitis 

DSS-induced acute or chronic colitis mouse model were prepared using 6-week-old C57BL/6 female mice (Samtako, Osan, Korea), as previously described [23]. To assess the induction of acute colitis, mice were subjected to 3% or 5% (*w*/*v*) dextran sodium sulfate (molecular weight: 36,000–50,000 kDa, MP Biomedicals, Santa ana, CA, USA) dissolved in drinking water given ad libitum, as illustrated in Figures 6A (Left) and 7A. The DSS solutions were made freshly per 2 days. Control non-DSS-fed mice had access to sterile distilled water. The humane endpoint (euthanasia required) for body weight loss is 20% (as compared to the original body weight of an animal). Body weight loss may not exceed 20% without an approved Exception request. 

### 2.16. Clinical Score and Histology

For clinical score of colitis, body weight, occult or gross blood lost per rectum, and stool consistency were determined every other day during the colitis induction. The clinical score was assessed by two trained investigators blinded to the treatment groups [23]. For immunohistochemistry of tissue sections, mouse distal colon tissues were fixed in 10% formalin and embedded in paraffin. Paraffin sections (4 μm) were cut and stained with hematoxylin and eosin (H&E). Histopathologic score and a semi-quantitative approach were used (H-score), in which a board-certified pathologist (Dr. Min-Kyung Kim, Kim Min-Kyung Pathology Clinic, Seoul, Korea) independently scored each organ section without prior knowledge of the treatment groups, as previously described [23,24].

### 2.17. Protein Purification and Mass Spectrometry

To identify Rubicon-binding proteins, BMDMs collected and lysed with NP-40 buffer (50 mM HEPES, pH 7.4, 150 mM NaCl, 1 mM EDTA, 1% (*v*/*v*) NP-40), supplemented with a complete protease inhibitor cocktail (Roche, Basal, Switzerland). Post-centrifuge supernatants were pre-cleared with protein A/G beads at 4 °C for 2 h. Pre-cleared lysates were subjected to immunoprecipitation with αRubicon for 18 h at 4 °C. Precipitates were washed extensively with lysis buffer. Proteins bound to beads were eluted and separated on a NuPAGE 4–12% Bis-Tris gradient gel (Life Technologies, Carlsbad, CA, USA). After silver staining (Life Technologies, Carlsbad, CA, USA), specific protein bands were excised and analysed by Ion Mobility Mass Spectrometer at Korea Basic Science Institute Mass Spectrometry facility, and amino acid sequences were determined by database searches.

### 2.18. Myeloperoxidase Activity Assay

To measure the activity of myeloperoxidase, we used myeloperoxidase activity assay kit (Abcam ab105136; Cambridge, UK). The colons of mice were homogenized and resuspended in MPO assay buffer. The samples were centrifuged at 13,000× *g* for 10 min and collect the supernatant. The MPO substrate was added with supernatant in 96-well plate. TNB solution was mixed and incubated 5 to 10 min. The plate was measured at 412 nM using MMR SPARK^®^ Microplate Reader (Männedorf, Switzerland). All assays were performed as recommended by the manufacturer.

### 2.19. Synthesis of Mito-TIPTP

We developed Mito-TIPTP, a p22phox N8 mimetic compound [15] conjugated with TPP, which is primarily used for mitochondrial targeting, are provided in the Supplementary Materials.

### 2.20. Statistical Analysis

All data were analyzed using Student’s *t*-test with Bonferroni adjustment or ANOVA for multiple comparisons, and are presented as mean ± SD. Statistical analyses were conducted using the SPSS (Version 12.0) statistical software program (SPSS, Chicago, IL, USA). Differences were considered significant at *p* < 0.05. For survival, data were graphed and analyzed by the product limit method of Kaplan and Meier, using the log-rank (Mantele-Cox) test for comparisons using GraphPad Prism (version 5.0, La Jolla, CA, USA).

## 3. Result

### 3.1. Rubicon Localizes to the Cytosol and Mitochondria

**Figure 1:**
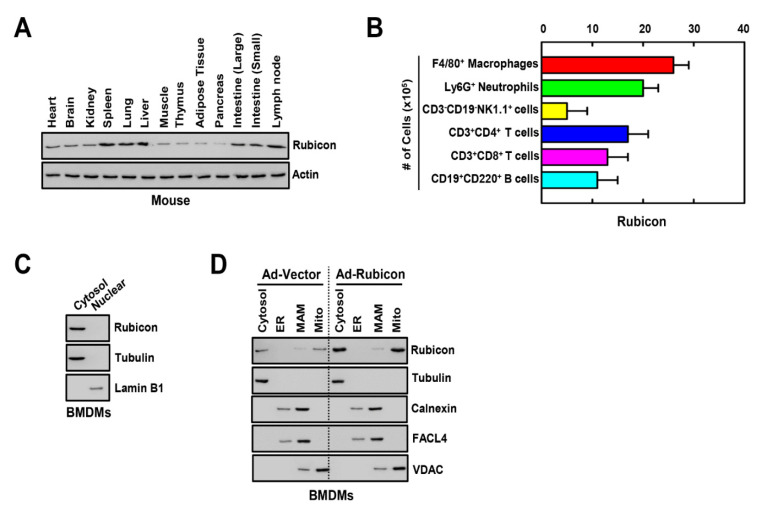
Tissue and subcellular distribution of Rubicon. (**A**) Each tissue was separated from mouse and homogenized. Rubicon expression was assessed by immunoblotting (IB) in various tissues from C57BL/6 normal mice. Whole-cell lysates (WCL) were used for the IB with αActin. (**B**) Cell frequencies were determined with the 10-color flow cytometry panel and centralized manual gating on cryopreserved splenocytes samples. Differences in the frequency of major splenocytes subsets. (**C**) BMDMs were nuclear and cytoplasmic fractions separated and analyzed for Rubicon by IB. αTubulin was detected as cytoplasmic protein loading controls. αLamin B1 was detected as nuclear loading controls. (**D**) BMDMs from WT mice were transduced with Ad-Rubicon or Ad-Vector (MOI = 10) for 2 days. BMDMs were subcellularly fractionated and subjected to IB with αRubicon. Levels of tubulin (cytosolic), calnexin (endoplasmic reticulum (ER) and mitochondria-associated membrane (MAM)), fatty acid CoA ligase 4 (FACL4, MAM) and voltage-dependent anion channels (VDAC, mitochondrial) protein in each fraction were determined by IB. Data shown are representative of three independent experiments with similar results (**A**,**C**,**D**). Data shown are the means ± SD of five experiments (**B**).

Previous studies indicate that Rubicon is ubiquitously expressed in tissues [25], and we found that Rubicon was strongly expressed in spleen, lung, liver, intestines, and lymph nodes in mice (Figure 1A). To determine the cellular distribution of Rubicon in the spleen, we analyzed the number of Rubicon-expressing cells in various immune cell types using FACS. Macrophages and neutrophils showed a higher population of Rubicon compared with NK, T, and B cells in splenocytes (Figure 1B). Next, we evaluated the subcellular localization of Rubicon in BMDMs. Cellular fractionation data showed that Rubicon was located in the cytosol but not the nucleus (Figure 1C). Next, to identify the detailed localization of Rubicon in the cytosol, we fractionated the cytosol into four components, including the cytosol, endoplasmic reticulum, mitochondria-associated membrane, and mitochondria. Rubicon was localized in the cytosol and mitochondria in WT or Rubicon-overexpressing BMDMs (Figure 1D).

### 3.2. Rubicon Interacts with P22phox in the Mitochondrial Outer Membrane

**Figure 2:**
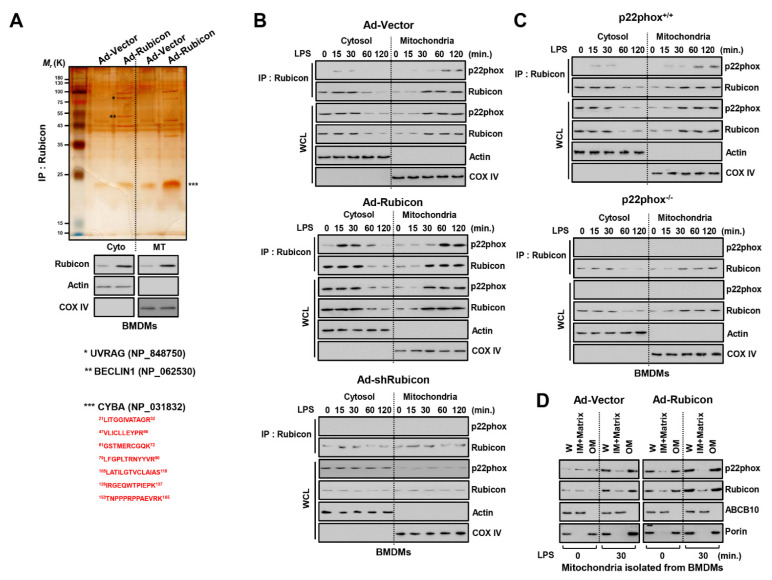
Rubicon−mediated p22phox translocated to mitochondria. (**A**) Identification of p22phox as endogenous binding partners of Rubicon in mitochondria. BMDMs were transduced with Ad−Rubicon or Ad-Vector (MOI = 10) for 2 days, followed by nuclear and cytoplasmic fractions separated and subjected to IP with αRubicon. Binding partners were confirmed by silver staining and mass spectrometric analysis (up). BMDMs were subcellularly fractionated and subjected to IB with αRubicon. αTubulin was detected as cytoplasmic protein loading controls. αLamin B1 was detected as nuclear loading controls (middle). The red-colored letters indicate the p22phox (CYBA) peptides identified from mass spectrometry analysis (down). (**B**,**C**) BMDMs were transduced with Ad-Rubicon, Ad−shRubicon, or Ad−Vector (MOI = 10) for 2 days (**B**) or BMDMs from p22phox^+/+^ and p22phox^−/−^ (**C**) and stimulated with LPS (100 ng/mL) for indicated times, followed by IP with αRubicon and IB with αp22phox. WCLs were used for IB with αRubicon, αp22phox, αCOX IV, and αActin. (**D**) Mitochondria isolated from BMDMs were subjected to digitonin extraction to separate the outer-membrane fraction (OM) and the fraction containing the inner membrane and matrix (IM + Matrix). These fractions and whole mitochondria (W) were subjected to IB using αRubicon. Mitochondrial VDAC/porin (an outer membrane protein) and ABCB10 (an inner-membrane protein). Data shown are representative of three independent experiments with similar results.

In our previous study, Rubicon was a binding partner for p22phox and enhanced cytosolic ROS levels in macrophages. This interaction was essential for upregulating cytosolic ROS upon inflammation or microbial infection in macrophages [13]. To determine whether Rubicon interacts with p22phox in mitochondria, we fractionated WT or Rubicon-overexpressing BMDMs and THP-1 cells and performed co-immunoprecipitation with Rubicon followed by mass spectrometry. Several directly associated proteins were identified, including autophagy-related proteins, such as UV radiation resistance-associated gene protein (UVRAG, 78K), BECLIN1 (52K), and p22phox (cytochrome b-245 light chain, CYBA, 21K) in both the cytosolic and mitochondrial fractions of macrophages (Figure 2A and Figure S1A). Next, we treated cells with LPS and prepared cytosolic and mitochondrial fractions to examine the endogenous binding of Rubicon and p22phox in BMDMs. In the cytosol, this interaction was primarily identified within 15 and 30 min in the cytosol, and 60 and 120 min in the mitochondria. Furthermore, we found that the interaction of Rubicon-p22phox was strongly maintained for 8 h in the mitochondria (Figure 2B and Figure S1B). We assumed that the Rubicon-p22phox complex was translocated from the cytosol to the mitochondria following an inflammatory stimulus. Moreover, in Rubicon-overexpressing BMDMs, the Rubicon-p22phox interaction was enhanced in the cytosol and mitochondria but declined in Rubicon-knockdown BMDMs. Interestingly, knockdown of Rubicon decreased the stability of p22phox in both fractions and reduced the translocation of p22phox to the mitochondria (Figure 2B). We also determined whether p22phox deficiency mediated the association of Rubicon-p22phox and the stability of Rubicon. Rubicon was slightly decreased in p22phox-deficient BMDMs, but translocation to the mitochondria was still evident (Figure 2C). We monitored the mitochondrial binding sites in Rubicon by preparing fractions from GST-Rubicon-overexpressing 293T cells. GST-Rubicon deletions of 505–557, 567–625, or 625–760 decreased translocation from the cytosol to the mitochondria, suggesting that the 505–760 region is essential for translocation to mitochondria (Figure S1C). Next, we investigated the location of p22phox and Rubicon in the mitochondrial membrane. We isolated mitochondria from BMDMs and examined Rubicon and p22phox by separating them from the outer and inner membrane. Rubicon and p22phox were located in the outer membrane in the mitochondria, and overexpression of Rubicon enhanced p22phox levels in the mitochondria (Figure 2D).

Previous work has demonstrated that Rubicon, in association with the Beclin1-VPS34- UVRAG-containing Class III PI(3)K complex, as a molecule required for LC3-associated phagocytosis (LAP) but not autophagy against bacteria [26,27]. To examine whether Rubicon was related to LAP in LPS treatment, we measured the level of LC3 conjugated GFP in RAW 264.7 macrophages. In starvation, the level of LC3 was reduced in Ad-Rubicon but increased in Ad-shRubicon. In LPS treatment, there is no difference in intensity of LC3 among the three groups of macrophages (Figure S2A). Additionally, the formation of LC3^+^ phagosome and the level of proteins related to LAP is not dependent in Rubicon (Figure S2B,C). Thus, the Rubicon-p22phox interaction results in translocation to the mitochondrial outer membrane through a mitochondrial targeting domain and is essential for the stability of p22phox in the mitochondria.

### 3.3. Interaction of Rubicon-p22phox Enhances Both Cytosolic ROS and mtROS and Reduces Mitochondrial Activity and Biogenesis

**Figure 3:**
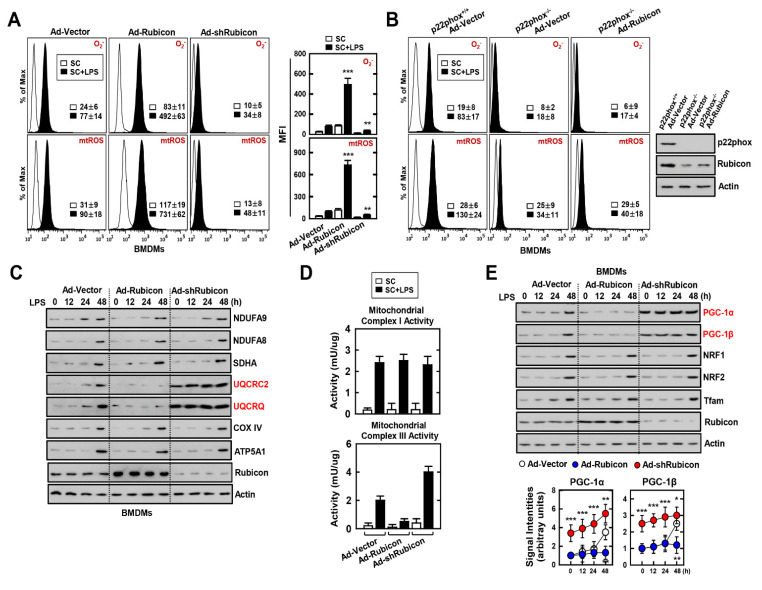
Effects of Rubicon–p22phox interaction in the mitochondrial activity and biogenesis. (**A**,**B**) BMDMs were transduced with Ad-Rubicon, Ad-shRubicon, or Ad-Vector (MOI = 10) for 2 days and stimulated with LPS (100 ng/mL) for 30 min. FACS analysis for superoxide (up) and mitoROS (down). Quantitative analysis of mean fluorescence intensities of ROS (box). p22phox and Rubicon expression by IB (**B**, right). (**C**) BMDMs was stimulated with LPS (100 ng/mL) for indicated times and subjected to IB with αNDUFA9, αNDUFA8, αSDHA, αUQCRC2, αUQCRQ, αCOX IV, αATP5A1, αRubicon, and αActin. (**D**) Mitochondrial Complex I (up) and III (down) Activity. (**E**) BMDMs was stimulated with LPS (100 ng/mL) for the indicated times and subjected to IB with αPGC-1α, αPGC-1β, αNRF1, αNRF2, αTfam, αRubicon, and αActin (up) and signal intensity of PGC-1α and PGC-1β were measured in indicated times (down). Data shown are the means ± SD of three experiments (**A**,**B**,**D**). Data shown are representative of five independent experiments with similar results (**C**,**E**). Statistical significance was determined by Student’s *t*-test with Bonferroni adjustment (* *p* < 0.05; ** *p* < 0.01; *** *p* < 0.001) compared with Ad-Vector.

Rubicon is an important activator of cytosolic ROS by inducing the NADPH oxidase complex following an inflammatory response [13,26]. We previously studied the intimate relationship between Rubicon and p22phox in the upregulation of cytosolic ROS [13]. Surprisingly, we found that the Rubicon/p22phox interaction occurred not only in the cytosol but also in the mitochondria (Figure 2). Next, we investigated the role of Rubicon and p22phox in the induction of cytosolic ROS and mtROS following inflammation in BMDMs. The relative increase in both ROS was significantly higher in Rubicon-overexpressing BMDMs, whereas the levels of cytosolic ROS and mtROS were slightly augmented in Rubicon-knockdown BMDMs (Figure 3A). Furthermore, p22phox-deficient BMDMs exhibited decreased ROS levels compared with WT BMDMs. There was no increase in ROS levels in p22phox-deficient BMDMs overexpressing Rubicon, suggesting that both p22phox and Rubicon are required for cytosolic ROS and mtROS production (Figure 3B). Excessive ROS attenuates mitochondrial function and biogenesis by disrupting the oxidative phosphorylation (OXPHOS) system [28]. To evaluate mitochondrial activity and biogenesis, we confirmed the expression of the OXPHOS complex subunits. Interestingly, during LPS treatment, cytochrome bc1 complex subunit 2 (UQCRC2) and cytochrome bc1 complex subunit 8 (UQCRQ), which are subunits of complex III, were decreased in Rubicon-overexpressed BMDMs but increased in Rubicon-knockdown BMDMs (Figure 3C). In addition, the activity of mitochondrial complex III showed similar results depending on Rubicon expression in LPS-treated BMDMs (Figure 3D). To determine whether Rubicon is associated with mitochondrial biogenesis, we measured the level of proteins involved in mitochondrial biogenesis, including peroxisome proliferator-activated receptor gamma coactivator (PGC)-1α, PGC-1β, nuclear respiratory factor (NRF) 1, NRF2, and mitochondrial transcription factor A (Tfam). Interestingly, PGC-1α and PGC-1β were significantly increased, but not NRF1, NRF2, or Tfam (Figure 3E). These results indicate that the interaction of Rubicon-p22phox upregulates ROS levels in the cytosol and mitochondria, and Rubicon inhibits mitochondrial activity and biogenesis by downregulating the expression of mitochondrial complex III subunits as well as PGC-1α and PGC-1β.

### 3.4. Rubicon Inhibits Mitochondrial Metabolism by Upregulating Glycolysis

**Figure 4:**
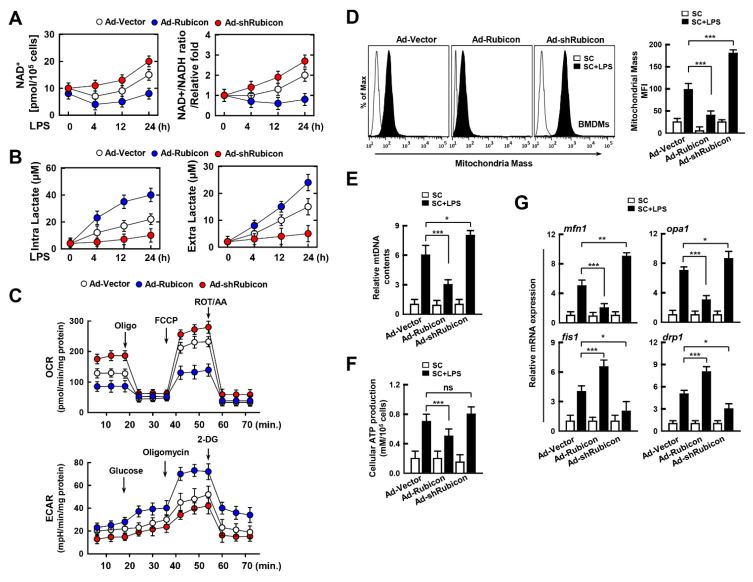
Effects of Rubicon in mitochondrial metabolism. BMDMs were transduced with Ad-Rubicon, Ad-shRubicon, or Ad-Vector (MOI = 10) for 2 days and stimulated with LPS (100 ng/mL) for indicated times. (**A**) NAD^+^/NADH ratio. (**B**) Lactate production. (**C**) The real-time measurement of OCR was analyzed by sequential treatment with oligomycin, FCCP, and rotenone/Actinomycin A, as an indicator of oxidative metabolism (left) or ECAR were analyzed by sequential treatment with glucose, oligomycin, and 2-DG, as an indicator of glucose oxidation. (**D**) Mitotracker fluorescence signals were assessed by a flow cytometric analysis. (Left) Representative histograms from seven independent replicates. (Right) Bar graph indicates the mitochondrial mass mean fluorescence intensities (MFIs). (**E**) Mitochondrial DNA (mtDNA) content in BMDMs measured by quantitative real-time PCR. The mtDNA content was normalized to nuclear DNA. (**F**) Cellular ATP production. (**G**) Quantitative real-time PCR of fusion or fission genes. Statistical significance was determined by Student’s *t*-test with Bonferroni adjustment (* *p* < 0.05; ** *p* < 0.01; *** *p* < 0.001) compared with Ad-Vector. Data shown are representative of seven independent experiments with similar results. Results are expressed as means ± SD of seven experiments.

Mitochondria are the fundamental sub-organelle for cellular metabolism. Activation of the immune response by immune cells is directly related to cellular metabolism, which is enhanced by glycolysis, followed by the production of inflammatory cytokines and chemokines, as well as ROS levels for inflammation [29,30]. As Rubicon may affect mitochondrial metabolism, we examined the role of Rubicon in cellular metabolism in LPS-treated BMDMs. First, we measured the amount of NAD^+^ and the ratio of NAD^+^/NADH in Rubicon-overexpressing or knockdown BMDMs. Rubicon decreased the NAD^+^ levels and the NAD^+^/NADH ratio, which suggests that Rubicon may inhibit mitochondrial metabolism (Figure 4A). Furthermore, we examined the levels of intra- and extra-lactate since the upregulation of glycolysis is associated with increased glycolytic metabolism. Upregulation of Rubicon enhanced both the production of intra- and extra-lactate, whereas knockdown of Rubicon decreased the production of lactate in LPS-treated BMDMs (Figure 4B). To determine the relationship between Rubicon and the metabolic state in real-time, we performed an extracellular flux analysis of the BMDMs. We monitored the OCR and ECAR every 5 min following treatment with mitochondrial complex inhibitors or ionophores such as oligomycin (Oligo), FCCP, rotenone/antimycin A (ROT/AA), and 2-DG. Consistent with previous data, overexpression of Rubicon attenuated mitochondrial metabolism, whereas Rubicon-knockdown resulted in the opposite effect. Also, extracellular acidification levels were increased in Rubicon-overexpressing BMDMs (Figure 4C). Furthermore, we examined the quality of mitochondria through measurement of mitochondrial mass, DNA, ATP production, and mRNA related to mitochondrial dynamics, including mfn1, opa1, fis1, and drp1. The mitochondrial mass was decreased in Ad-Rubicon but increased in Ad-shRubicon (Figure 4D). the contents of mtDNA and generation of cellular ATP also showed similar patterns of mitochondrial mass (Figure 4E,F). The expressions of mfn1 and opa1 associated mitochondrial fusion were declined, but the levels of fis1 and drp1 were increased in overexpressed-Rubicon BMDMs. However, the expression of fusion proteins was upregulated, while the level of fission proteins was downregulated in Ad-shRubicon BMDMs (Figure 4G). Thus, Rubicon decreases mitochondrial metabolism and quality, accompanied by reductions in the mitochondrial metabolism, mass, and dynamics in BMDMs.

### 3.5. Mito-TIPTP Enhances Mitochondrial Function through Inhibition of the Rubicon-p22phox Interaction

**Figure 5:**
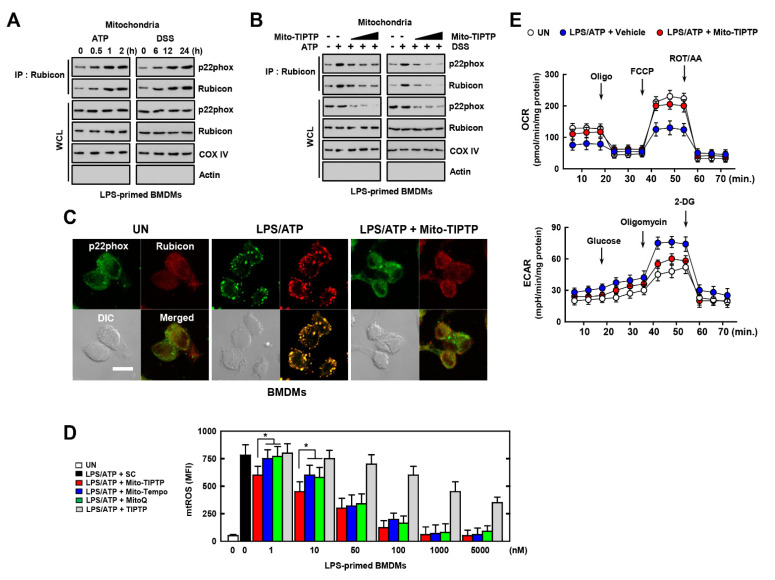
Effects of Mito−TIPTP in mitochondrial functions. BMDMs were mitochondria fractionated by Mitochondria Isolation Kit. (**A**) LPS (100 ng/mL)-primed mitochondria fractionated−BMDMs were activated with ATP (1 mM) for 30 min or DSS (3%) for 18 h, and subjected to IP with αRubicon. IB with αp22phox and αRubicon. WCLs were used for IB with αRubicon, αp22phox, αCOX IV, and αActin. (**B**) The experimental conditions follow the same pattern as outlined in (**A**). LPS−primed BMDMs were treated with Mito−TIPTP (1, 10, and 100 nM) for 1 h, and then activated with ATP for 30 min or DSS for 18 h. (**C**) Immunostaining of BMDMs treated with LPS/ATP in presence or absence of Mito−TIPTP (10 nM) and then immunolabeled with antibody to p22phox (Alexa Fluor 488) or Rubicon (Alexa Fluor 568). Scale bar, 10 μm. (**D**) FACS analysis for mitoROS of BMDMs treated with LPS/ATP in the presence or absence of Mito−TIPTP, Mito−Tempo, or MitoQ in indicated concentrations. Quantitative analysis of mean fluorescence intensities of ROS (box). (**E**) The real−time measurement of OCR, as an indicator of oxidative metabolism (left) or ECAR, as an indicator of glucose oxidation. Data shown are representative of seven independent experiments with similar results (**A**–**E**). Statistical significance was determined by Student’s *t*−test with Bonferroni adjustment (* *p* < 0.05) compared with solvent control (0.1% DMSO).

Colitis is a well-known inflammatory disease which triggers severe inflammation in the intestine. Excessive ROS production and the activation of the NLRP3 inflammasome is accompanied by the upregulation interleukin (IL)-1β and IL-18 [31,32]. As colitis is associated with activation of the NLRP3 inflammasome, we used ATP- or DSS-activated macrophages to examine the interaction between Rubicon and p22phox in vitro. Activation of LPS-treated BMDMs by ATP or DSS increased the interaction of Rubicon and p22phox in a time-dependent manner (Figure 5A). In our previous study, we developed inhibitors of the p22phox–Rubicon interaction in the cytosol, namely, TIPTP, as a potential therapeutic agent for RA through blocking the Rubicon–p22phox interaction and decreasing cytosolic ROS levels [15]. To examine the effect of TIPTP in a colitis model, we tested TIPTP with additional chemical moiety in BMDMs. To increase the specificity of TIPTP for targeting mitochondria, we added a molecule called TPP, which is primarily used for mitochondrial targeting [33]. We designated this compound, Mito-TIPTP, which exhibits a higher capacity to target mitochondria compared with TIPTP (data not shown). To examine the effect of Mito-TIPTP on the Rubicon–p22phox interaction, we administered Mito-TIPTP to LPS-primed BMDMs with ATP or DSS. Mito-TIPTP decreased the binding of Rubicon to p22phox in a dose-dependent manner in LPS-primed BMDMs (Figure 5B). We also observed a decrease in the co-localization of Rubicon-p22phox by Mito-TIPTP in mitochondria by confocal imaging (Figure 5C and Figure S3A). Furthermore, we measured the mtROS levels following treatment with various concentrations of Mito-TIPTP, other antioxidants of mtROS (Mito-Tempo and MitoQ), and antioxidants of cytosolic ROS (TIPTP) in LPS-primed BMDMs activated by ATP. At low doses (1~10 nM), Mito-TIPTP caused a significant decrease in mtROS levels compared with the other mtROS inhibitors. At high concentrations (>50 nM), Mito-TIPTP decreased the mtROS in a dose-dependent manner, but showed no significant difference compared with mtROS inhibitors (Figure 5D and Figure S3B,C). Remarkably, Mito-TIPTP had an IC_50_ of 0.05 μM, which is a 100-fold improvement in IC_50_ compared with that of TIPTP, which had an IC_50_ value of 5 μM (Figure 5D). We performed an extracellular flux analysis to determine whether Mito-TIPTP recovered mitochondrial metabolism in LPS-primed BMDMs stimulated by ATP. Mito-TIPTP increased mitochondrial metabolism and decreased the level of extracellular lactate in BMDMs (Figure 5E). Altogether, Mito-TIPTP attenuates mtROS levels by inhibiting the Rubicon–p22phox interaction and recovers mitochondrial metabolism.

### 3.6. Mito-TIPTP Alleviates Acute and Chronic DSS-Induced Colitis in Mice

**Figure 6:**
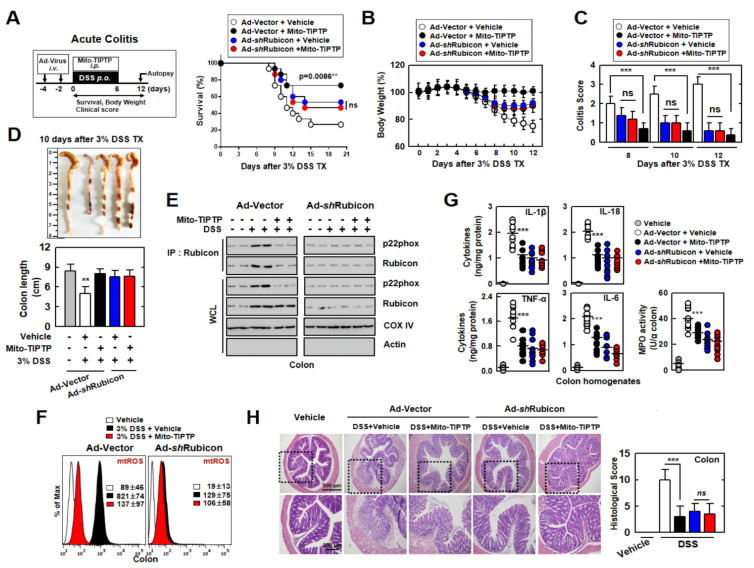
Mito−TIPTP has a therapeutic effect against acute DSS−induced colitis in mice. (**A**) Schematic of the acute colitis model transduced with Ad−vector or Ad−shRubicon virus and treated 3% DSS with Mito−TIPTP (50 ng/kg) (left). The survival of mice was monitored for 21 days; mortality was measured for *n* = 15 mice per group (right). (**B**) Weight loss (*n* = 8). (**C**) Colitis scores were obtained from clinical parameters (weight loss, stool consistency, and bleeding) (*n* = 8). (**D**) Image (up) and length (down) of colon in 3% DSS−treated mice with vehicle or Mito−TIPTP (*n* = 8). (**E**) Colon was used for IP with αRubicon, followed by IB with αp22phox and αRubicon. WCLs were used for IB with αp22hox, αRubicon, αActin, and αCOX IV. (**F**) FACS analysis for mtROS from colon (*n* = 8). (**G**) levels of cytokines and MPO activity in colon homogenates (*n* = 10). (**H**) Representative imaging of H&E staining of the colon (left) (*n* = 10). Histopathology scores were obtained from H&E staining, as described in methods (Materials and Methods) were determined in 3% DSS−treated mice with vehicle or Mito−TIPTP. Scale bar, 500 μm. Statistical significance was determined by Student’s *t*−test with Bonferroni adjustment (** *p* < 0.01; *** *p* < 0.001) compared with vehicle.

Next, we examined the therapeutic effects of Mito-TIPTP in acute and chronic DSS-induced colitis mouse models. We generated Rubicon-knockdown mice through adenoviral infection and treated the mice with DSS and Mito-TIPTP for 12 days. Mito-TIPTP increased the survival rate in WT mice against DSS-induced colitis but not in Ad-shRubicon-infected mice. Interestingly, decreased Rubicon delayed the mortality of mice, and we suspected that the inhibition of Rubicon was related to the regulation of the lethal immune response (Figure 6A and Figure S4A). The loss of body weight also decreased by approximately 20% in WT mice treated with Mito-TIPTP compared with untreated mice. In Rubicon-knockdown mice, there was no significant difference in body weight, but it was higher than untreated WT mice (Figure 6B and Figure S4B). The colitis score in the mice was significantly decreased in WT mice treated with Mito-TIPTP (Figure 6C and Figure S4C). After 10 days, we examined colon length, which is an indicator of colitis. In WT mice, colon length was recovered in Mito-TIPTP-treated mice, whereas, in Ad-shRubicon-infected mice, colon length was increased, but there was no effect of Mito-TIPTP (Figure 6D). Furthermore, we examined the binding of Rubicon and mtROS levels in the colon. The Rubicon-p22phox interaction was only increased in DSS-treated mice colon and decreased in Ad-shRubicon mice (Figure 6E). The mtROS levels were also reduced in WT mice treated with Mito-TIPTP but not in Ad-shRubicon mice (Figure 6F). We also measured the production of cytokines including IL-1β, IL-18, TNF-α, and IL-6, as well as the activity of myeloperoxidase (MPO), which is involved in the activation of inflammation (Figure 6G). H&E staining and immunohistochemistry revealed that the untreated colon impaired the function and loss of goblet cells by DSS in WT mice, but the addition of Mito-TIPTP recovered the colon barrier and goblet cells. Additionally, Rubicon-knockdown increased the viability of the colon, independent of Mito-TIPTP. The pathology scores for Rubicon and p22phox in the colon were increased in the untreated Ad-vector mice (Figure 6H and Figure S5).

**Figure 7:**
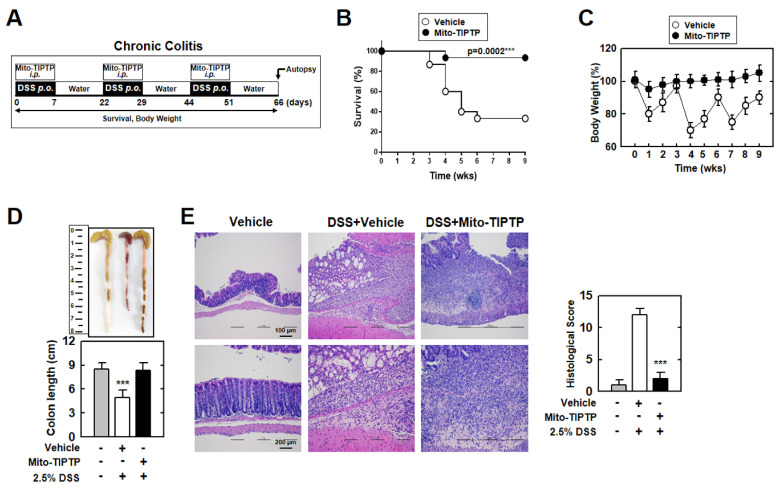
Mito−TIPTP alleviates chronic DSS−induced colitis in mice. (**A**) Schematic of the chronic colitis model treated 2.5% DSS with Mito−TIPTP (50 ng/kg). (**B**) The survival of mice was monitored for 9 weeks; mortality was measured for *n* = 15 mice per group. (**C**) Weight loss of vehicle or Mito−TIPTP in mice (*n* = 15). (**D**) Image (up) and length (down) of colon in 2.5% DSS−induced chronic colitis mice with vehicle or Mito−TIPTP. (**E**) Representative imaging of H&E staining of the colon (left) (*n* = 8). Histopathology scores were obtained from H&E staining were determined in 2.5% DSS−induced chronic colitis mice with vehicle or Mito−TIPTP. Scale bar, 100 μm. Statistical significance was determined by Student’s *t*−test with Bonferroni adjustment (*** *p* < 0.001) compared with vehicle.

Next, to examine the therapeutic effect of Mito-TIPTP against chronic colitis, we repeatedly exposed mice to DSS and Mito-TIPTP for 66 days (Figure 7A). Similar to acute colitis, Mito-TIPTP increased the survival of mice by approximately 60% (Figure 7B). Body weight fluctuated in the untreated mice, but Mito-TIPTP-treated mice maintained body weight (Figure 7C). Additionally, the colons of the Mito-TIPTP-treated mice were recovered, compared with mouse colons treated with only DSS, which were significantly damaged (Figure 7D). H&E imaging of the colon revealed that Mito-TIPTP significantly increased colon viability and goblet cells in DSS-treated mice (Figure 7E). Altogether, Mito-TIPTP exhibits a therapeutic effect against DSS-induced colitis by blocking the Rubicon–p22phox interaction in vivo.

### 3.7. Novel Biomarkers of Patients with Ulcerative Colitis

**Figure 8:**
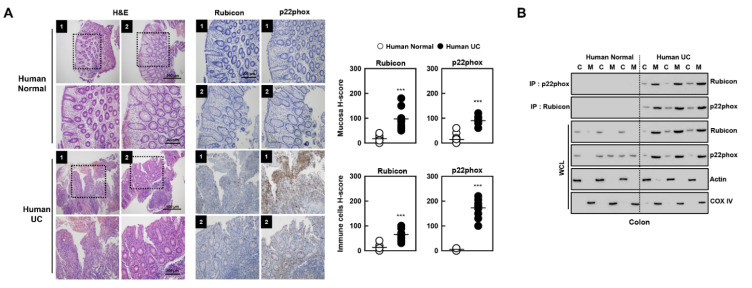
H&E staining and immunohistochemistry in colon in human normal and ulcerative colitis (UC) patients. (**A**) Human normal and UC patients were used for H&E staining and IHC with αp22phox and αRubicon (left). H-score Rubicon and p22phox in mucosa and immune cells in colon were calculated by multiplying the percentage of the stained area by the staining intensity (right). Representative images from five independent healthy controls and patients are shown. Insets, enlargement of outlined areas. Biological replicates (*n* = 10) for each condition were performed. (**B**) Colon of human normal and UC patients were cytoplasmic and mitochondria fractions separated and analyzed for Rubicon, subjected to IP with αRubicon and αRubicon. IB with αp22phox and αRubicon. WCLs were used for IB with αRubicon, αp22phox, αCOX IV, and αActin. Data from three of ten normal human and UC patients are shown. Statistical significance was determined by Student’s *t*-test with Bonferroni adjustment (*** *p* < 0.001) compared with human normal.

We further examined colonic Rubicon and p22phox expression between healthy controls and patients with ulcerative colitis (Figure 8A). Rubicon and p22phox expression were weakly positive in the cytosol of mucosal and immune cells from the normal colon; however, ulcerative colitis exhibited a strong positivity (H-score > 100). Furthermore, Rubicon interacted with p22phox primarily in the mitochondria of colon lysates from ulcerative colitis subjects, but not healthy controls (Figure 8B). These results indicate that the expression and interaction of Rubicon and p22phox in the colon are clinically significant in ulcerative colitis, suggesting that they may serve as biomarkers for ulcerative colitis.

## 4. Discussion

ROS are essential factors for inducing the inflammatory milieu, and they are produced in the cytosol and mitochondria. Rubicon is known as a trigger, which increases cytosolic ROS by interacting with p22phox in previous studies [13,14,15]. However, whether this interaction regulates the production of mtROS is unknown. In this study, we demonstrated the association of Rubicon-p22phox in the production of mtROS and the therapeutic effects of Mito-TIPTP against colitis. The main findings of this study were as follows: (1) the Rubicon–p22phox complex is translocated to the outer mitochondrial membrane; (2) the Rubicon–p22phox interaction enhances both cytosolic ROS and mtROS; (3) Rubicon inhibits mitochondrial metabolism and biogenesis; (4) Mito-TIPTP increases mitochondrial function by alleviating the binding of Rubicon to p22phox; and (5) Mito-TIPTP increases the survival of colitis-induced mice by blocking Rubicon–p22phox binding and restoring the mitochondria.

Rubicon is a well-known regulator of autophagy and the immune response by interacting with a variety of proteins. Noncanonical autophagy, also known as LAP, is triggered by the recognition of extracellular ligands binding to membrane receptors, such as the TLRs and Fc receptors (FcRs) [25]. Autophagic factors are recruited to generate a LAP-engaged-phagosome (LAPosome), which is a single membrane enveloping the pathogen. Rubicon is associated with the complex containing Beclin1, Vps34, UVRAG, and p150, to produce PI3P in the LAPosome membrane. Rubicon was also associated with p22phox and NOX2, activating NADPH oxidase and increasing cytosolic ROS. Rubicon-p22phox-NOX2 is recruited and embedded into the LAPosome membrane, which increases the generation of ROS to fuse the lysosome with LAPosome [34]. Furthermore, Rubicon regulates the immune response by interacting with 14–3-3β CARD9, which is necessary for the activation of pro-inflammatory signals through the formation of an M-CBM complex with BCL10 and MALT1, which is dependent on Dectin-1 or RIG-1 signaling [35,36]. We previously examined the regulation of NADPH oxidase by Rubicon, which interacts with p22phox and NOX2 and activates the complex to produce cytosolic ROS [13]. In monocyte-derived dendritic cells (Mo-DC), p22phox interact with NOX5, which increases the expression in differentiation in outer mitochondrial membrane. The NOX5-p22phox complex activates inflammation by regulating the JAK/STAT/MALK and NF-κB pathways [37]. In the present study, we found interactions between Rubicon and p22phox not only in the cytosol but also in the outer mitochondrial membrane. This interaction was associated with the generation of mitochondrial ROS accompanied by mitochondrial dysfunction. The Rubicon–p22phox complex appears to be translocated from the cytosol to the mitochondria in LPS-activated macrophages. These results suggested that Rubicon is an important protein for the production of ROS in both the cytosol and mitochondria.

In the cytosol, the NADPH oxidase complex, which is comprised of NOX2 (gp91phox), p67phox, p47phox, p22phox, p40phox, and Rac, is well-known for the production of O_2_^−^ and the conversion of NADPH to NADP^+^. The expression of NOX2 is increased upon stimulation, and the activity of NADPH oxidase is also upregulated. Increased ROS activates immune signaling and enhances the production of cytokines and chemokines [38,39]. mtROS were once considered by-products of normal mitochondrial metabolism, but current studies have demonstrated that mtROS has beneficial and detrimental roles in cell physiology and pathology. The generation of mtROS primarily occurs through the electron transport chain in the inner mitochondrial membrane during oxidative phosphorylation. Electron leaks from the mitochondrial electron transport chain react with oxygen, resulting in highly reactive free radicals, such as superoxide anion [40]. Several studies have shown that ROS influences cellular metabolism. Following inflammatory stimuli, ROS induces the activity of AMPK, which is a central mediator of cellular metabolism, including glycolysis, lipid metabolism, mitochondrial metabolism, cell growth, and autophagy [41]. ROS increases the phosphorylation of AMPK through the upregulation and release of Ca^2+^ and Ca^2+^/calmodulin-dependent protein kinase β, which subsequently modulates the AMP/ATP ratio [42]. Furthermore, the expression of hypoxia-induced factor-1α, which is a core activator for the expression of glycolytic genes, is upregulated by ROS. Enhanced glycolytic metabolism upregulates the pentose phosphate pathway, which generates NADPH [43,44]. We showed that the overexpression of Rubicon is linked to the upregulation of cytosolic and mitochondrial ROS in LPS-primed BMDMs. Excessive mtROS causes a decline in mitochondrial respiration and biogenesis. Specifically, the activity of mitochondrial complex III is disrupted in Rubicon-overexpressing macrophages. The expression of PGC-1α and 1β are decreased, but not other factors related to mitochondrial biogenesis. However, it is unclear how Rubicon regulates only PGC-1α and 1β, not other factors. We speculated that Rubicon may interact with upstream factors related to expression of PGC-1α and 1β, which is independent NRF 1/2 and Tfam, and a profound study is needed to understand this mechanism. Thus, this suggests that the increase in mtROS through the Rubicon–p22phox interaction alleviates the mitochondrial metabolism and immunometabolism of immune cells. Further studies of the relationship between Rubicon and immunometabolism will be important in the context of inflammatory disease.

As excessive ROS commonly occurs in inflammatory diseases, targeting the regulation of cytosolic or mitochondrial ROS is important for treatment. Previously, we found that the regulation of Rubicon is a potential treatment strategy for sepsis and RA. The p22phox-derived peptide, which interacts with Rubicon, decreased NOX2 activity and increased the production of inflammatory cytokines in LPS-treated macrophages or CLP-induced mice by blocking the interaction of p22phox and Rubicon. Furthermore, the regulation of p22phox–Rubicon significantly augmented the survival of mice against sepsis, suggesting that control of ROS through the inhibition of p22phox–Rubicon is crucial for sepsis treatment [14]. We developed TIPTP as a p22phox inhibitor and evaluated its effects in RA mice and patient cells. In RA mice, the Rubicon–p22phox interaction increased the level of ROS and inflammation, followed by severe pathogenesis. TIPTP recovered the inflammatory response and pathogenesis by reducing the association of p22phox and Rubicon in RA mice and patients [15].

We focused on the interaction of p22phox–Rubicon in mitochondria for colitis treatment. IBDs are severe chronic diseases accompanied by a hyper-inflammatory response in the gastrointestinal tract. Excessive inflammation damages the intestinal mucosal barrier and disrupts homeostasis, facilitating the invasion of pathogens. Abnormalities in the intestinal mucosal barrier may cause pathogenesis and the development of IBDs [1,45,46]. Abnormalities in the intestinal mucosal barrier resulted from increased intestinal permeability, altered tight junction composition, defective homeostasis of commensal bacteria composition, and uncontrolled host-innate and adaptive immunity in colon has been associated with pathogenesis and the development of IBD [47]. In a mice colitis model, IL-18 signaling induced the breakdown of barrier integrity and reduced goblet cell maturation in the intestine [48]. IL-17 firmly amplified TNF-α, CCL20, and IL-23 mRNA and increased the activation of NF-κB, p38, and Erk pathways in intestinal neuroendocrine and goblet cells in human IBD [49]. Furthermore, over-activated inflammation induces high oxidative stress, which causes cellular damage [1]. Rubicon is increased in a variety of inflammatory diseases, and we also identified its upregulation and increased binding with p22phox in colitis-induced in vivo models and in colitis patients (Figure 8). Furthermore, we developed a therapeutic agent for colitis based on the Rubicon–p22phox interaction by adding TPP to TIPTP, designated Mito-TIPTP, which increased the targeting efficacy to the mitochondria. TPP is a commonly used molecule for mitochondrial-targeted agents used in anti-cancer and antifungal therapy. TPP contains a lipophilic cation, which can achieve a high uptake efficiency and accumulation in the mitochondrial membrane. These characteristics are useful for antioxidants targeted to the mitochondria [33]. There are some antioxidants that are conjugated with TPP, such as ubiquinone, plastoquinone, MitoQ, MitoQH2, and SKQ2 [50,51,52,53]. MitoQ is a well-known mitochondrial-targeted antioxidant, which has a similar function as Coenzyme Q_10_. It mediates lipid peroxidation, production of ROS, and apoptosis by its scavenging activity [51]. Furthermore, MitoQ showed clinical effectiveness in Parkinson’s disease, hepatitis C infection, and vascular disease [54,55,56]. As a MitoQ, Mito-TIPTP may exhibit antioxidant activity by targeting the p22phox–Rubicon interaction. We examined the marked antioxidant effect of Mito-TIPTP compared with MitoQ and Mito-Tempo against colitis. Mitochondrial function was also increased following the treatment of Mito-TIPTP in colitis along with diminished mtROS and inflammatory cytokine production.

Here, we show that the p22phox–Rubicon interaction enhances mtROS in the outer mitochondrial membrane by disrupting mitochondrial function. These results indicate that Rubicon is a fundamental regulator of cellular and mitochondrial ROS in inflammatory disease. We designed Mito-TIPTP to inhibit the interaction of p22phox–Rubicon in colitis. Mito-TIPTP showed a significant therapeutic effect against colitis; thus, Mito-TIPTP represents a potential therapeutic agent for colitis.

## Data Availability

The data is contained within the article or supplementary material.

## Supplementary Materials

The following are available online at https://www.mdpi.com/2076-3921/10/12/1954/s1, Figure S1: Rubicon interacts with p22phox in mitochondria; Figure S2: Rubicon is not required for LC3-associated phagocytosis following LPS stimulation; Figure S3: Mito-TIPTP alleviates the level of mtROS; Figure S4: Mito-TIPTP decreases the susceptibility against DSS-induced colitis; Figure S5: Mito-TIPTP upregulates viability of colon in DSS-induced colitis; Figure S6: Full-length images of the blots; Table S1: Experimental procedures for the synthesis of Mito-TIPTP (4).

## Abbreviations

Rubicon	Run/cysteine-rich-domain-containing Beclin1-interacting autophagy protein
NADPH	Nicotinamide adenine dinucleotide phosphate
ROS	Reactive oxygen species
mtROS	Mitochondrial ROS
LPS	Lipopolysaccharide
BMDMs	Bone marrow-derived macrophages
ATP	Adenosine triphosphate
DSS	Dextran sulfate sodium
IBDs	Inflammatory bowel diseases
TIPTP	2-(tetrahydroindazolyl)phenoxy-*N*-(thiadiazolyl) propenamide 2
CLP	Cecal ligation procedure
TPP	Triphenylphosphonium
LAP	LC3-associated phagocytosis
OXPHOS	Oxidative phosphorylation
UQCRC2	Cytochrome bc1 complex subunit 2
UQCRQ	Cytochrome bc1 complex subunit 8
PGC	Peroxisome proliferator-activated receptor gamma coactivator
NRF	Nuclear respiratory factor
Tfam	Mitochondrial transcription factor A
OCR	Oxygen consumption rate
ECAR	Extracellular acidification rate
Oligo	Oligomycin
FCCP	Carbonyl cyanide-p-trifluoromethoxy-phenylhydrazone
ROT/AA	Rotenone/antimycin A
2-DG	2-deoxy-d-glucose
IL-1β	Interleukin-1β
TLRs	Toll-like receptors
FcRs	Fc receptors
Mo-DC	Monocyte-derived dendritic cells
AMPK	5′ adenosine monophosphate-activated protein kinase
IB	Immunoblotting
IP	Immunoprecipitation
ELISA	Enzyme-linked immunosorbent assay
H&E staining	hematoxylin and eosin staining
