# Correction: Kim et al. Mito-TIPTP Increases Mitochondrial Function by Repressing the Rubicon-p22phox Interaction in Colitis-Induced Mice. *Antioxidants* 2021, *10*, 1954

**DOI:** 10.3390/antiox12071415

**Published:** 2023-07-13

**Authors:** Jae-Sung Kim, Ye-Ram Kim, Sein Jang, Sang Geon Wang, Euni Cho, Seok-Jun Mun, Hye-In Jeon, Hyo-Keun Kim, Sun-Joon Min, Chul-Su Yang

**Affiliations:** 1 Department of Bionano Technology, Hanyang University, Seoul 04673, Republic of Korea; 2 Institute of Natural Science & Technology, Hanyang University, Ansan 15588, Republic of Korea; 3 Center for Bionano Intelligence Education and Research, Ansan 15588, Republic of Korea; tpdls26@hanyang.ac.kr (S.J.);; 4 Department of Molecular and Life Science, Hanyang University, Ansan 15588, Republic of Korea; 5 Department of Applied Chemistry, Hanyang University, Ansan 15588, Republic of Korea; 6 Department of Chemical & Molecular Engineering, Hanyang University, Ansan 15588, Republic of Korea

In the original publication [1], there was a mistake in Figure 7. Results figures from Figure 6D,H have been inserted in Figure 7. The corrected Figure 7 appears below.

The authors state that the scientific conclusions are unaffected. This correction was approved by the Academic Editor. The original publication has also been updated.

## Figures and Tables

**Figure 7:**
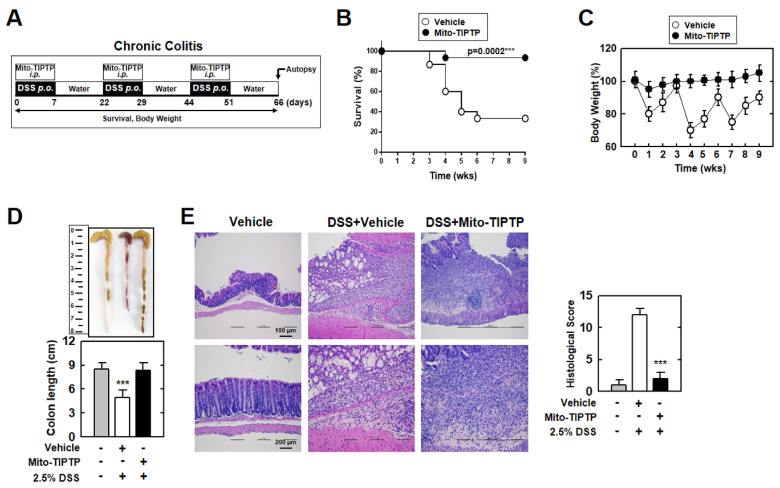
Mito−TIPTP alleviates chronic DSS−induced colitis in mice. (**A**) Schematic of the chronic colitis model treated 2.5% DSS with Mito−TIPTP (50 ng/kg). (**B**) The survival of mice was monitored for 9 weeks; mortality was measured for *n* = 15 mice per group. (**C**) Weight loss of vehicle or Mito−TIPTP in mice (*n* = 15). (**D**) Image (up) and length (down) of colon in 2.5% DSS−induced chronic colitis mice with vehicle or Mito−TIPTP. (**E**) Representative imaging of H&E staining of the colon (left) (*n* = 8). Histopathology scores were obtained from H&E staining were determined in 2.5% DSS−induced chronic colitis mice with vehicle or Mito−TIPTP. Scale bar, 100 μm. Statistical significance was determined by Student’s *t*−test with Bonferroni adjustment (*** *p* < 0.001) compared with vehicle.
