# Supplementary material for: Mito-TIPTP Increases Mitochondrial Function by Repressing the Rubicon-p22phox Interaction in Colitis-Induced Mice
Source: Antioxidants (Basel). 2021 Dec 6;10(12):1954. doi: 10.3390/antiox10121954 (PMC8750874; doi:10.3390/antiox10121954)
Supplement: Supplementary file 1 [file antioxidants-10-01954-s001.zip › antioxidants-1460513-supplementary.pdf]

Supplementary Materials

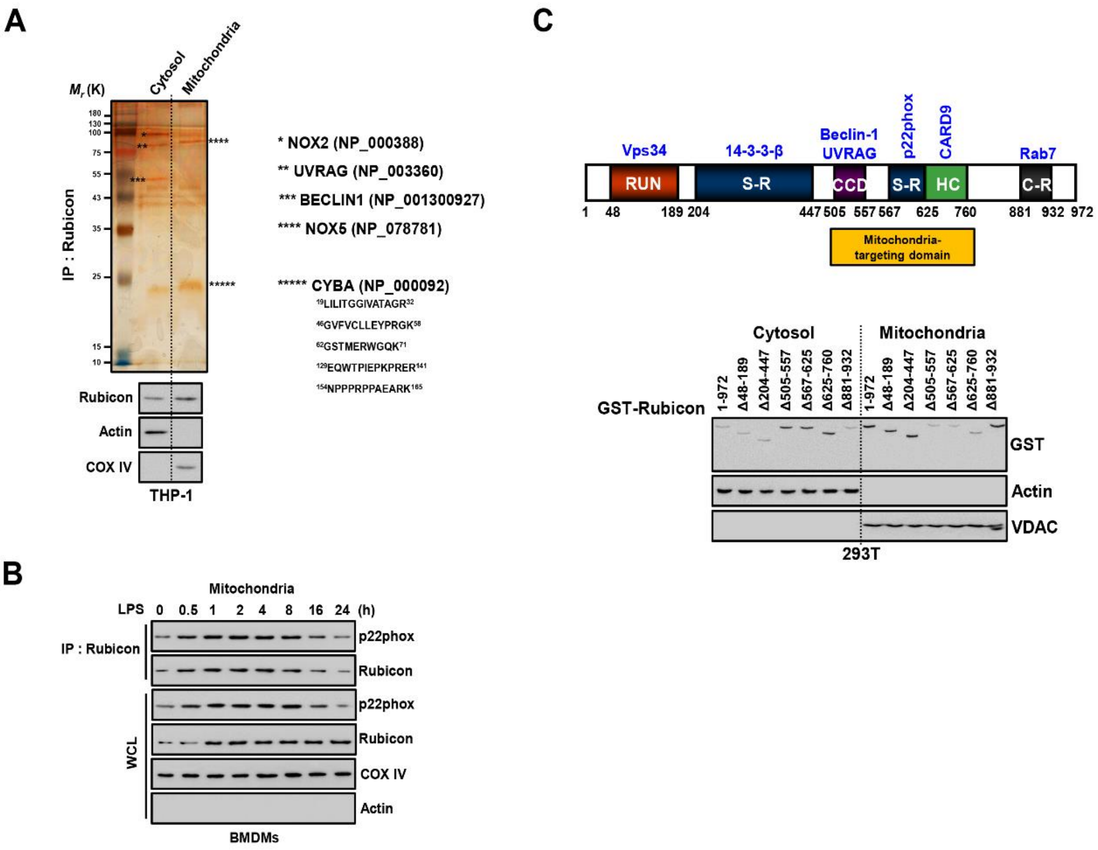

Figure S1. Rubicon interacts with p22phox in mitochondria; (**A**) THP-1 cells were fractionated for cytosol and mitochondria fraction. Each fraction was immunoprecipitated with αRubicon and analyzed by mass-spectrometry to identify the binding proteins of Rubicon. (**B**) BMDMs were stimulated with LPS (100 ng/ml) for indicated times, followed by mitochondria fractionated by Mitochondria Isolation Kit, and subjected to IP with αRubicon. IB with αp22phox and αRubicon. WCLs were used for IB with αRubicon, αp22phox, αCOX IV, and αActin. (**C**) Structure of Rubicon (up) and domain mapping of mitochondria targeting domain in Rubicon (down). GST-Rubicon or truncated mutant were transfected in 293T cells and fractionated for cytosol and mitochondria, followed by IB with αGST, αActin, and αVDAC. Data shown are representative of three independent experiments with similar results.

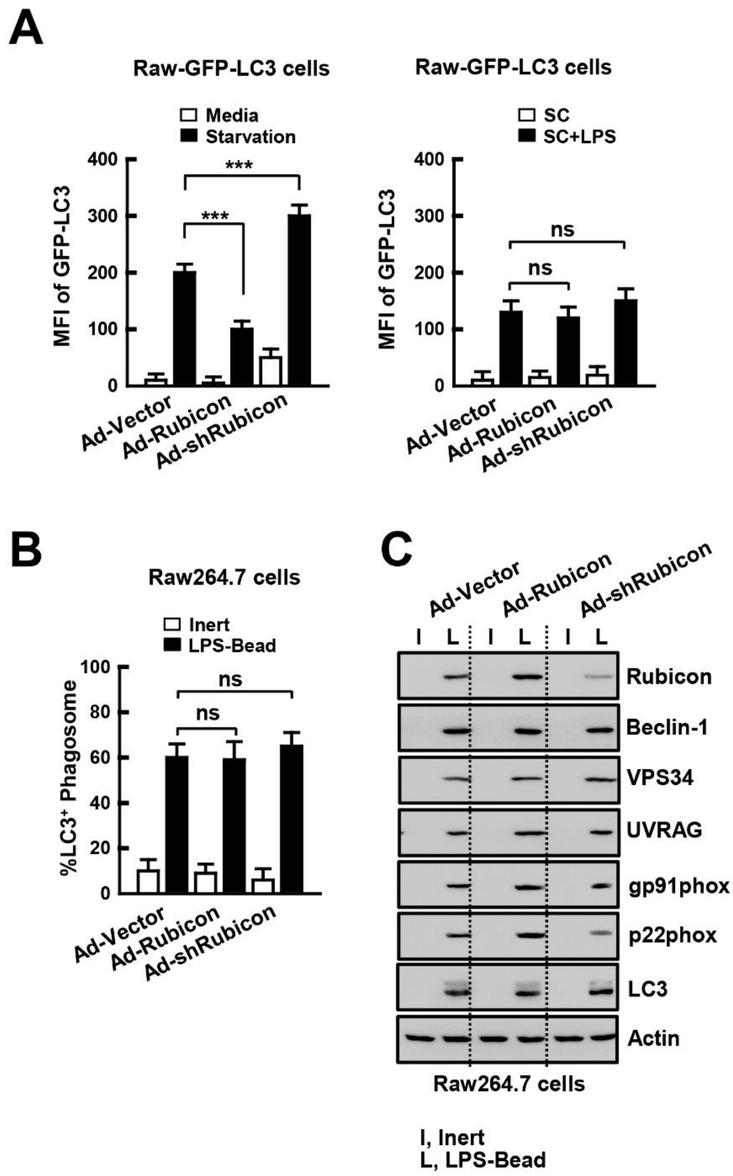

Figure S2. Rubicon is not required for LC3-associated phagocytosis following LPS stimulation. (**A**) Raw-GFP-LC3 cells or (**B** and **C**) Raw264.7 cells were transduced with Ad-Rubicon, Ad-shRubicon, or Ad-Vector (MOI = 10) for 2 days and stimulated with starvation for 6 h or LPS (100 ng/ml) for 30 min. (**A**) GFP–LC3 puncta were assessed at 18 h by confocal microscopy and flow cytometry. (**B** and **C**) Raw264.7 cells were fed inert beads or LPS-beads for 1 h. Immunofluorescent staining was performed for LC3B and analyzed by microscopy and percent of LC3+ phagosomes were calculated. Data are presented as mean ± SD (n=50 cells per genotype pooled from four independent experiments). (**C**) Phagosomes were purified using sucrose gradient procedures. Phagosome proteins were solubilized in SDS-PAGE and blotted with the indicated antibodies. The results presented are representative of three independent experiments. Statistical significance was determined by Student's *t*-test with Bonferroni adjustment (*** $p < 0.001$) compared with Ad-Vector.

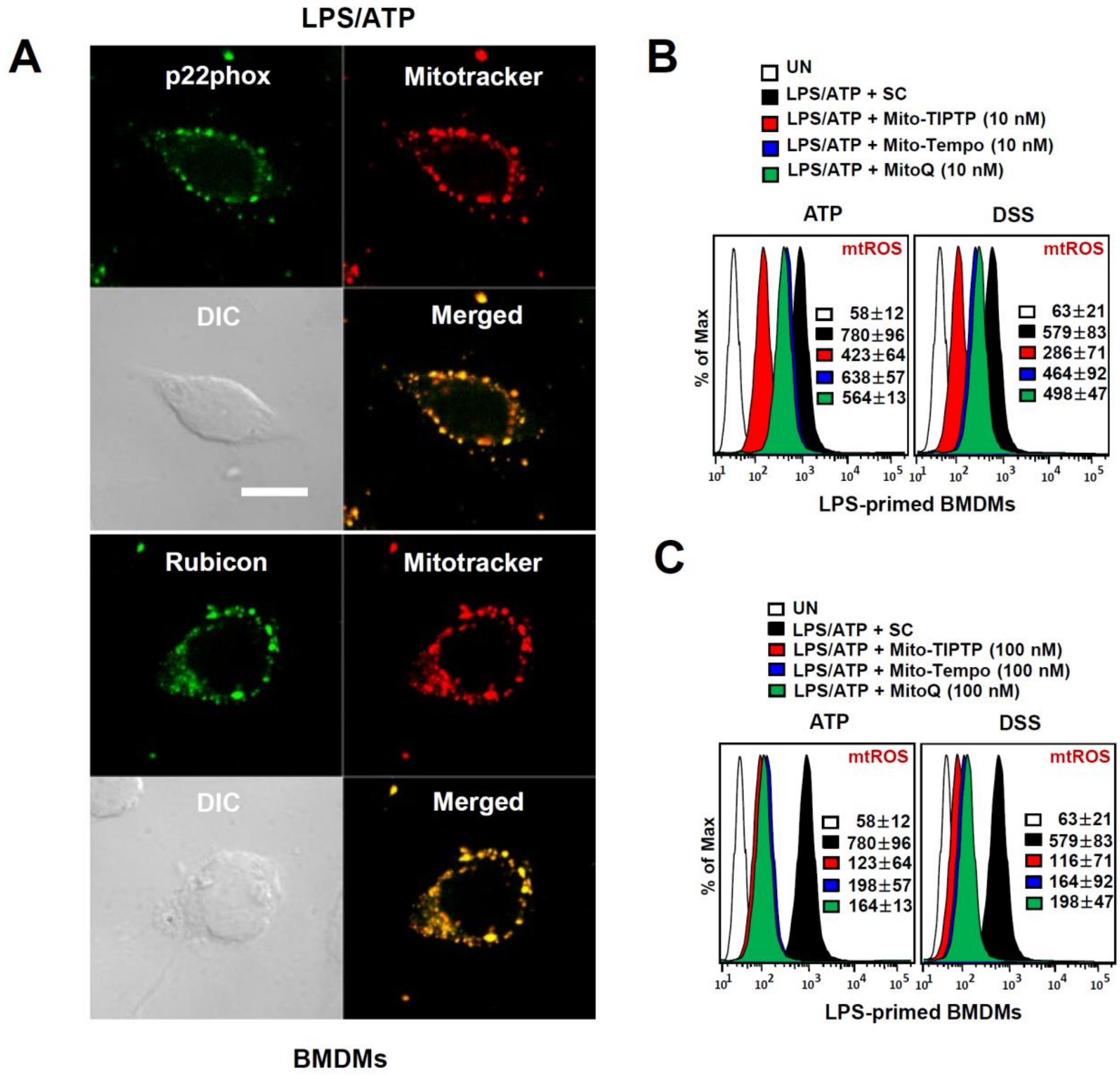

Figure S3. Mito-TIPTP alleviates the level of mtROS. (**A**) Confocal imaging of p22phox and Rubicon with mitochondria in LPS-primed BMDMs with ATP. (**B** and **C**) FACS analysis for mtROS from LPS-primed BMDMs with ATP (1 mM) or 3 % DSS and Mito-TIPTP, Mito-Tempo or MitoQ in indicated concentrations. Data shown are representative of three independent experiments with similar results. Data shown are the means ± SD of three experiments (**B** and **C**).

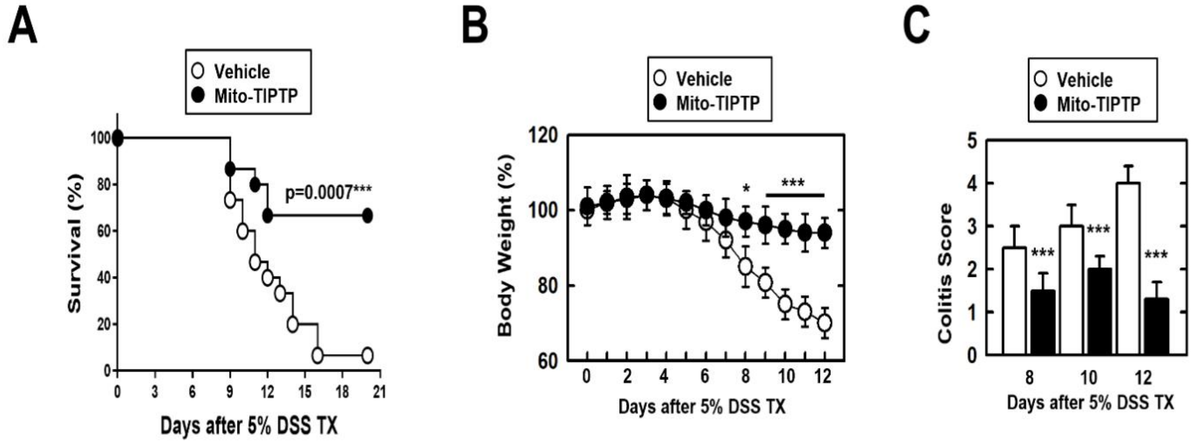

Figure S4. Mito-TIPTP decreases the susceptibility against DSS-induced colitis. (**A**) Mice treated 5% DSS with vehicle or Mito-TIPTP (50 ng/kg). The survival of mice was monitored for 21 days; mortality was measured for *n* = 15 mice per

group. (**B**) Weight loss of vehicle or Mito-TIPTP in 5% DSS treated mice (n = 10). (**C**) Colitis scores were obtained from clinical parameter (weight loss, stool consistency, bleeding) of indicated mice (n = 10). Statistical significance was determined by Student's t-test with Bonferroni adjustment (*** $p < 0.001$) compared with vehicle.

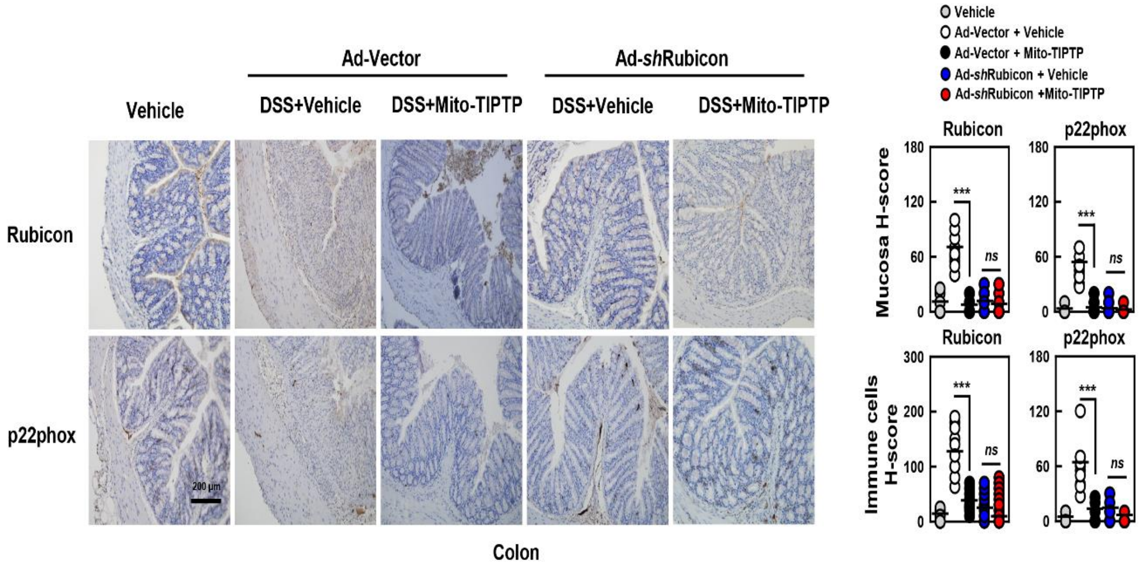

Figure S5. Mito-TIPTP upregulates viability of colon in DSS-induced colitis. Mice infected by Ad-Vector or Ad-shRubicon and treated 3 % DSS with vehicle or Mito-TIPTP (50 ng/kg) and analyzed by immunohistochemistry (IHC) with αp22phox and αRubicon (left). H-score Rubicon and p22phox in mucosa and immune cells in colon were calculated by multiplying the percentage of the stained area by the staining intensity (right). Statistical significance was determined by Student's t-test with Bonferroni adjustment (** $p < 0.01$; *** $p < 0.001$) compared with vehicle.

Fig 1A

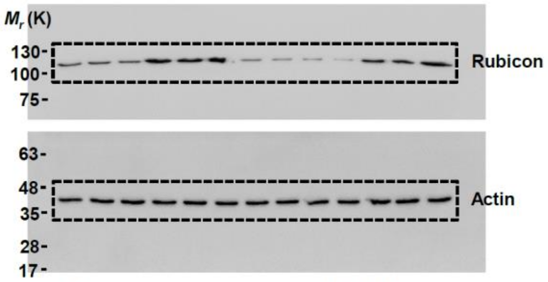

Fig 1C

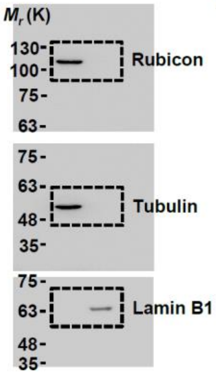

Fig 1D

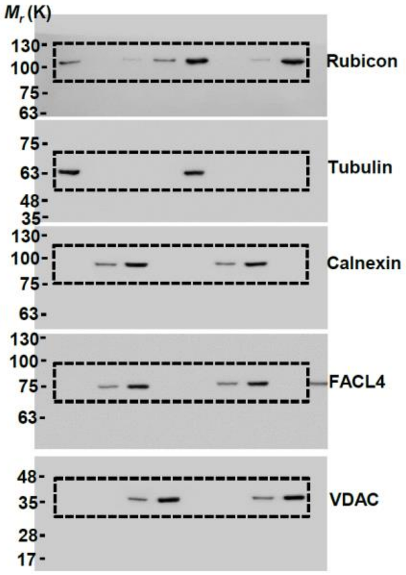

Fig 2A

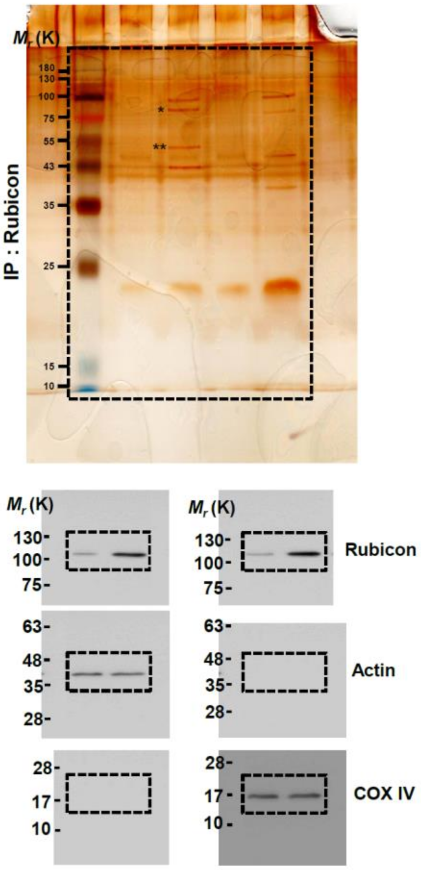

Fig 2B-up

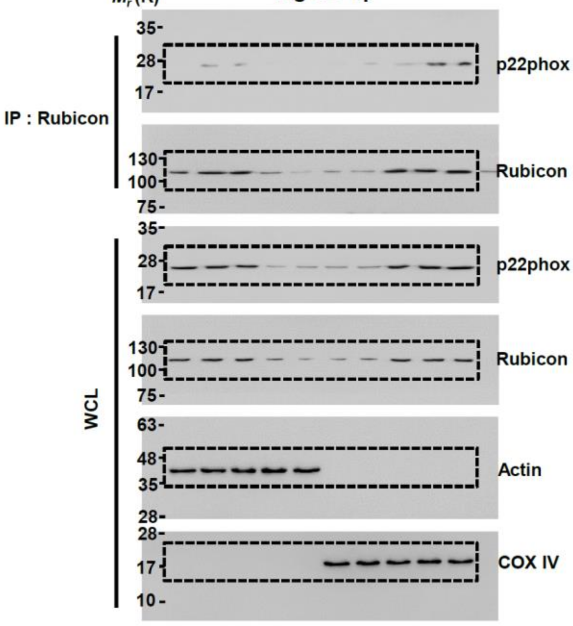

Fig 2B-middle

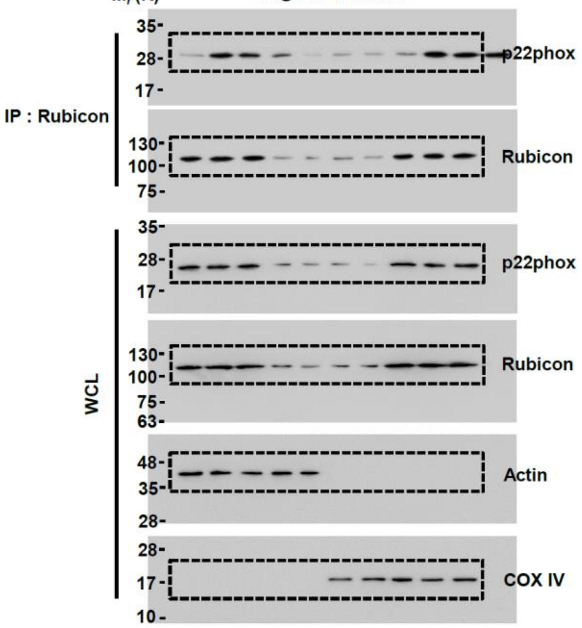

Fig 2B-down

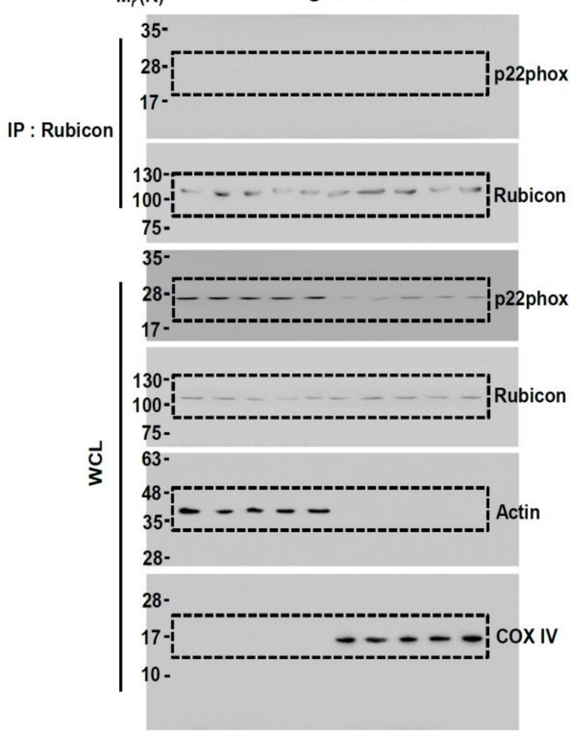

Fig 2C-up

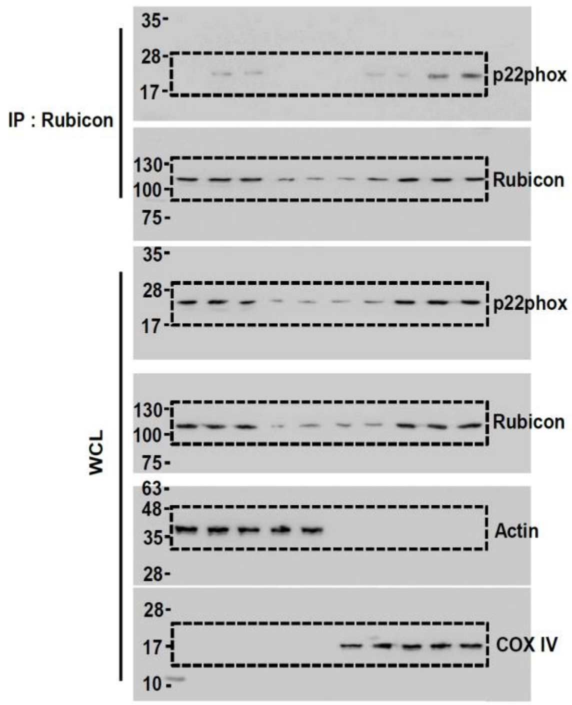

Fig 2C-down

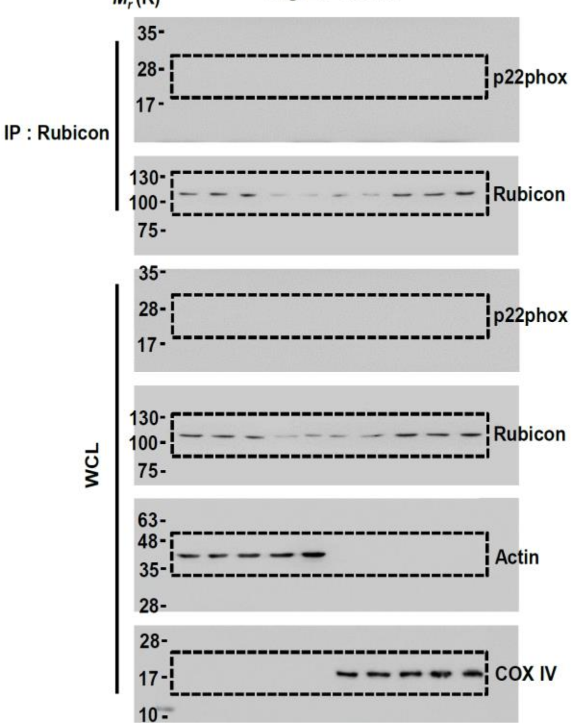

Fig 2D

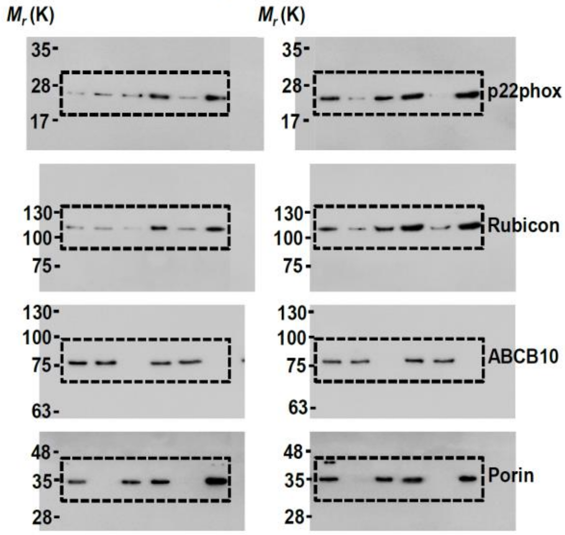

Fig 3C

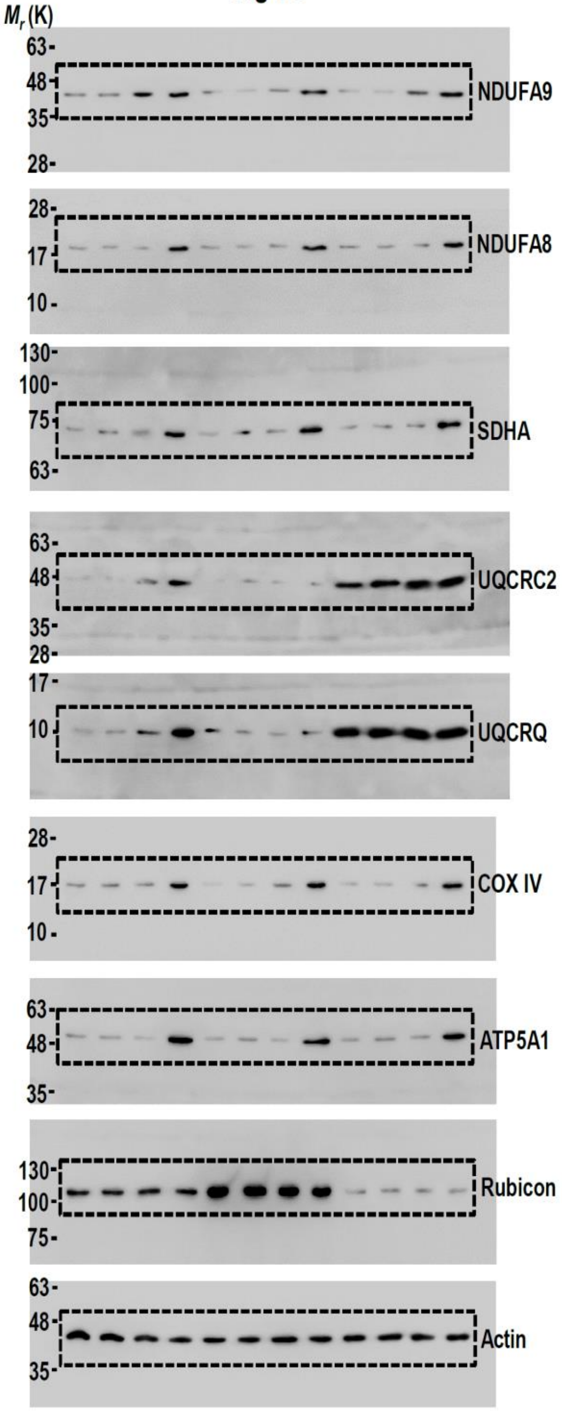

M_r (K)
63
48
35
28
NDUFA9
28
17
10
NDUFA8
130
100
75
63
SDHA
63
48
35
28
UQCRC2
17
10
UQCRQ
28
17
10
COX IV
63
48
35
ATP5A1
130
100
75
Rubicon
63
48
35
Actin

Fig 3E

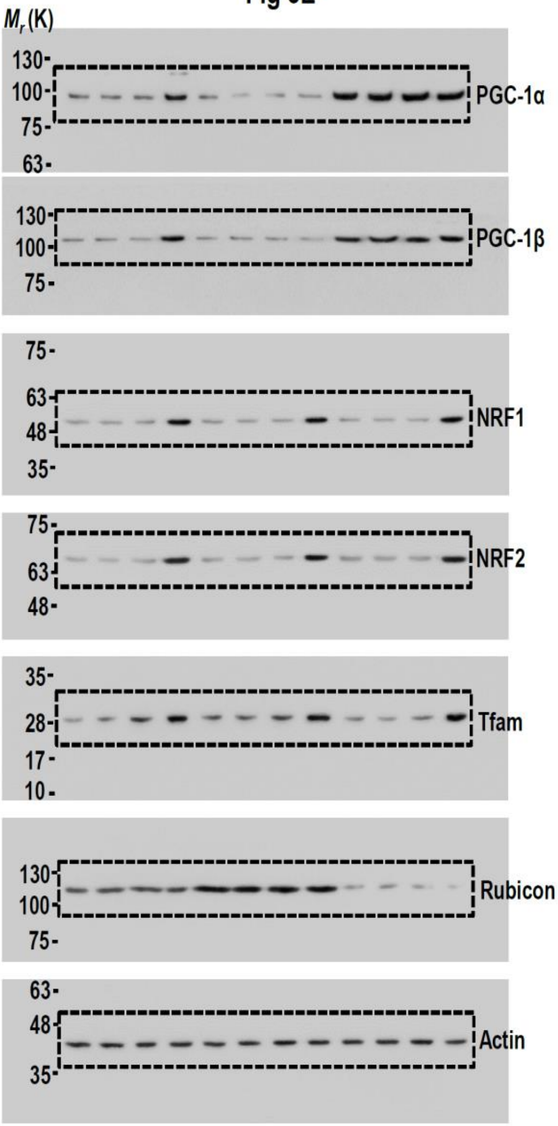

M_r (K)
130
100
75
63
PGC-1α
130
100
75
PGC-1β
75
63
48
35
NRF1
75
63
48
NRF2
35
28
17
10
Tfam
130
100
75
Rubicon
63
48
35
Actin

Fig 5A

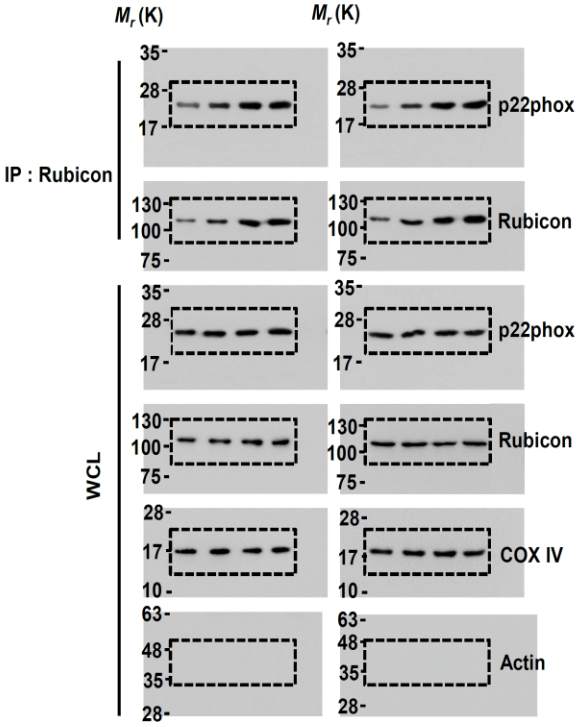

Fig 5B

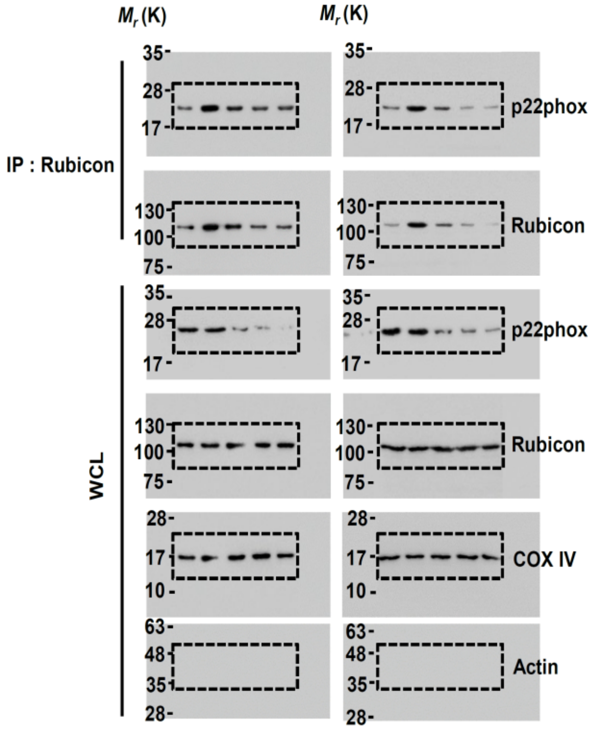

Fig 6E

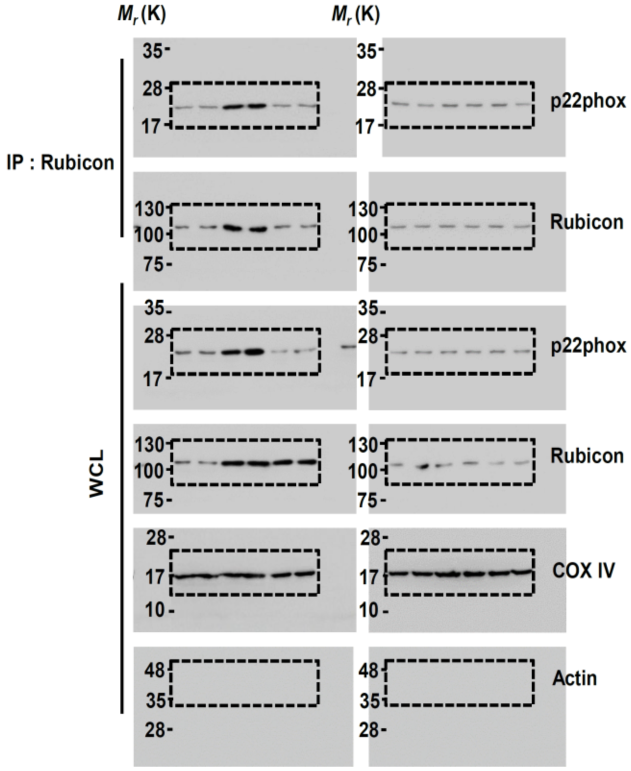

Figure S6. Full-length images of the blots.

Table S1. Experimental procedures for the synthesis of Mito-TIPTP (4).

General: All reactions were conducted using oven-dried glassware under an atmosphere of argon (Ar). All commercially available reagents and anhydrous solvents were obtained from Sigma Aldrich, TCI, Alfa, Junsei, Samchun, Dae-Jung Chemical and were used without further purification. Solvents CH_2Cl_2 was dried and distilled following usual protocols. Organic solvents were evaporated with reduced pressure using a rotary evaporator. Reactions were followed by TLC analysis using silica gel 60 F_{254} with fluorescent indicator using UV lamp and ninhydrin solution with heat as visualizing agents. Flash chromatography was carried out using Merck silica gel 60 (0.063–0.200 mm) and KANTO silica gel 60N (spherical, neutral). The ^{1}H NMR spectra and ^{13}C NMR spectra were measured with Bruker AVANCE III HD 400. ^{1}H NMR chemical shifts are expressed in parts per million (δ) downfield to $CHCl_3$ (δ = 7.26), ^{13}C NMR chemical shifts are expressed in parts per million (δ) relative to the central $CDCl_3$ resonance (δ = 77.0). Coupling constants in ^{1}H NMR are in Hz. The following abbreviations were used to designate multiplicities: s = singlet, d = doublet, t = triplet, q = quartet, dd = doublet of doublets, m = multiplet. $CDCl_3$ was used as NMR solvent and standard material TMS (tetramethylsilane) wasn't contained. LC/MS analyses were performed on Agilent 6125 SQ LCMS system.

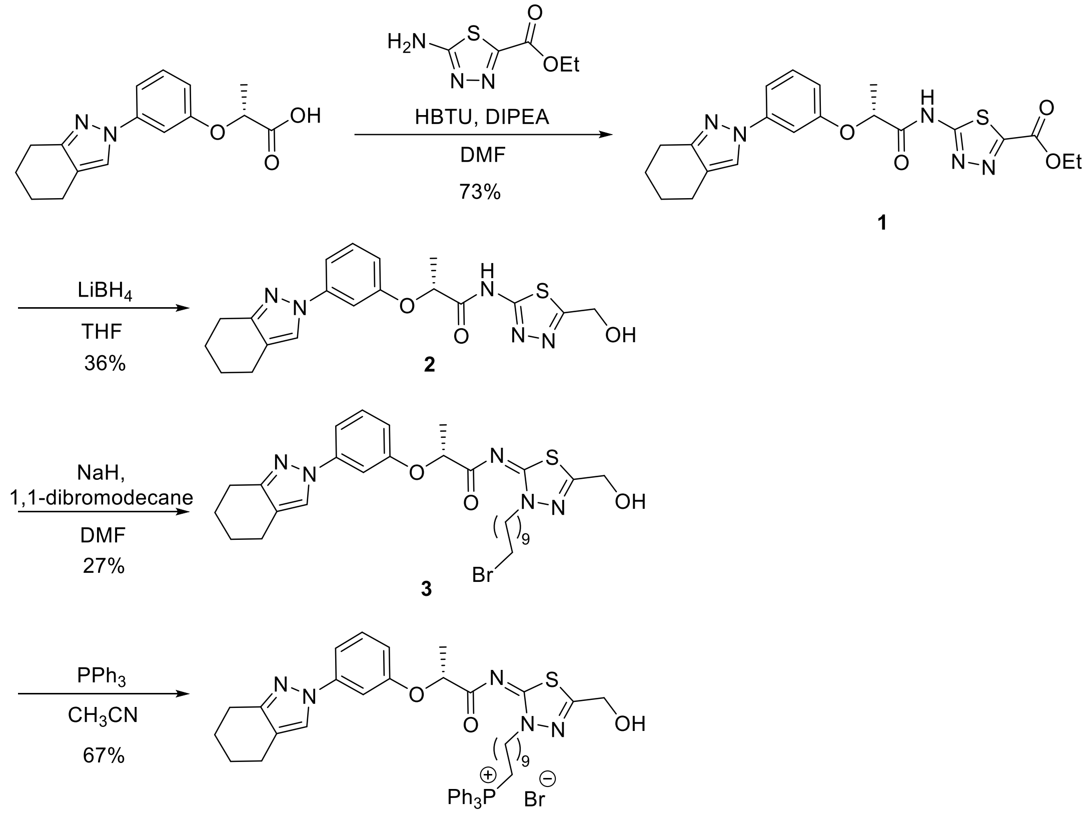

mito-TIPTP (4)

Scheme 1. Synthesis of Mito-TIPTP (4).

Ethyl (*R*)-5-(2-(3-(4,5,6,7-tetrahydro-2H-indazol-2-yl)phenoxy)propanamido)-1,3,4-thiadiazole-2-carboxylate (1).

To a solution of (*R*)-2-(3-(4,5,6,7-tetrahydro-2*H*-indazol-2-yl)phenoxy)propanoic acid [1] (143 mg, 0.499 mmol) in DMF (5.0 mL) was added *N,N*-diisopropylethylamine (442 μL, 2.54 mmol), HBTU (378 mg, 0.998 mmol) and ethyl 5-amino-1,3,4-thiadiazole-2-carboxylate (130 mg, 0.749 mmol). After the reaction mixture was stirred at 75 °C for 3 h, it was cooled to room temperature, quenched with water and extracted with dichloromethane (3 x 50 mL). The combined organic layers were washed with brine and concentrated under reduced pressure. The crude residue was purified by column chromatography on silica gel (hexane/ethyl acetate = 1:1) to give the title compound **1** (160 mg, 73%) as orange color powder. ^{1}H NMR (400 MHz, $CDCl_3$) δ 11.18 (s, 1H), 7.57 (s, 1H), 7.34 (s, 1H), 7.30 (t, *J* = 8.1, 1H), 7.22-7.21 (m, 1H), 6.78-6.76 (m, 1H), 5.14 (q, *J* = 6.5 Hz, 1H), 4.47 (t, *J* = 6.9 Hz 2H), 2.79 (t, *J* = 6.7 Hz, 2H), 2.63 (t, *J* = 6.3 Hz, 2H), 1.88-1.76 (m, 4H), 1.68 (d, *J* = 6.4 Hz, 3H), 1.41 (t, *J* = 6.8 Hz, 3H).

***R*)-N-(5-(hydroxymethyl)-1,3,4-thiadiazol-2-yl)-2-(3-(4,5,6,7-tetrahydro-2H-indazol-2-yl)phenoxy)propanamide (2)**

To a solution of ethyl (R)-5-(2-(3-(4,5,6,7-tetrahydro-2H-indazol-2-yl)phenoxy)propanamido)-1,3,4-thiadiazole-2-carboxylate **1** (160 mg, 0.342 mmol) in THF (3.6 mL) was added $LiBH_4$ (2.0M in THF) (0.543 mL, 1.09 mmol). After the reaction mixture was stirred at 25 °C for 12 h, it was quenched with saturated NH_4Cl solution and extracted with ethyl acetate (3 x 50 mL). The combined organic layers were washed with brine and concentrated under reduced pressure. The residue was purified by column chromatography (hexane/ethyl acetate = 1:3) on silica gel to give the title compound **2** (52 mg, 36%) as solid. ^1H NMR (400 MHz, $CDCl_3$) *δ* 7.52 (s, 1H), 7.27-7.25 (m, 1H), 7.23 (s, 1H), 7.13 (d, *J* = 7.6 Hz, 1H), 6.72 (d, *J* = 8.1 Hz, 1H), 5.11 (q, *J* = 6.5 Hz, 1H), 4.93 (s, 2H), 2.74 (t, *J* = 6.1 Hz, 2H), 2.58 (t, *J* = 5.9 Hz, 2H), 1.86-1.71 (m, 4H), 1.67 (d, *J* = 6.7 Hz, 3H). ^{13}C NMR (100 MHz, $CDCl_3$) δ 170.4, 166.8, 159.6, 157.4, 151.9, 141.5, 130.4, 124.5, 118.7, 112.5, 112.4, 107.6, 74.5, 59.4, 29.8, 23.4, 23.3, 20.6, 18.3. LRMS (ESI) *m/z*: $[M+H]^+$ calcd for $C_{19}H_{22}N_5O_3S$ 400.1; found 400.1.

(*R*,*E*)-*N*-(3-(10-bromodecyl)-5-(hydroxymethyl)-1,3,4-thiadiazol-2(3H)-ylidene)-2-(3-(4,5,6,7-tetrahydro-2H-indazol-2-yl)phenoxy)propenamide (3)

To solution (*R*)-N-(5-(hydroxymethyl)-1,3,4-thiadiazol-2-yl)-2-(3-(4,5,6,7-tetrahydro-2H-indazol-2-yl)phenoxy)propanamide **2** (52 mg, 0.130 mmol) in DMF (2.6 mL) was added sodium hydride (5.2 mg, 0.130 mmol, 60% dispersion in mineral oil), 1,10-dibromodecane (15.7 μL, 0.070 mmol). After the reaction mixture was stirred at 25 °C for 4 h, it was quenched with water and extracted with dichloromethane (3 x 30 mL). The combined organic layers were washed with brine and concentrated under reduced pressure. The crude residue was purified by column chromatography on silica gel (diethyl ether/hexane = 1:1) to give the title compound **3** (20 mg, 25%) as colorless oil. ^1H NMR (400 MHz, $CDCl_3$) *δ* 7.54 (s, 1H), 7.24-7.17 (m, 3H), 6.73 (dd, *J* = 10.1, 1.4 Hz, 1H), 5.04 (q, *J* = 6.7 Hz, 1H), 4.72 (m, 2H), 4.34 (m, 2H), 3.41 (t, *J* = 6.8 Hz, 2H), 2.74 (t, *J* = 6.4 Hz, 2H), 2.60 (t *J* = 6.3 Hz, 2H), 1.87-1.70 (m, 10H), 1.65 (d, *J* = 6.7 Hz, 3H), 1.44-1.36 (m, 2H), 1.25 (m, 8H). LRMS (ESI) m/z: $[M+H]^+$ calcd for $C_{29}H_{41}BrN_5O_3S$ 618.2; found 618.3.

(*R*,*E*)-(10-(5-(hydroxymethyl)-2-((2-(3-(4,5,6,7-tetrahydro-2H-indazol-2-yl)phenoxy)propanoyl)imino)-1,3,4-thiadiazol-3(2H)-yl)decyl)triphenylphosphonium bromide (4, mito-TIPTP)

To a solution of (*R*,*E*)-*N*-(3-(10-bromodecyl)-5-(hydroxymethyl)-1,3,4-thiadiazol-2(3*H*)-ylidene)-2-(3-(4,5,6,7-tetrahydro-2H-indazol-2-yl)phenoxy)propenamide **3** (20 mg, 0.0323 mmol) in CH_3CN (0.32 mL) was added triphenylphosphine (7.0 mg, 0.0258 mmol). The reaction mixture was stirred at 75 °C for 12 h using reflux. The crude mixture was washed with hexane and diethyl ether to remove impurity. The residue was dried under reduced pressure to give the mito-TIPTP **4** (19 mg, 67%) as white solid. ^1H NMR (400 MHz, $CDCl_3$) *δ* 7.82-7.65 (m, 15H), 7.48 (s, 1H), 7.16-7.12 (m, 3H), 6.67-6.64 (m, 1H), 5.02 (q, *J* = 6.7 Hz, 1H), 4.89-4.80 (m, 2H), 4.35-4.27 (m, 2H), 3.68-3.53 (m, 2H), 2.71 (t, *J* = 6.2 Hz, 2H), 2.57 (t, *J* = 6.0 Hz, 2H), 1.83-1.70 (m, 8H), 1.61 (d, *J* = 6.7 Hz, 3H), 1.24-1.13 (m, 4H), 1.03 (m, 8H). LRMS (ESI) m/z: $[M]^+$ calcd for $C_{47}H_{55}N_5O_3PS$ 800.3; found 800.4.

References

1. Kim, Y. R.; Kim, J. S.; Gu, S. J.; Jo, S.; Kim, S.; Young Kim, S.; Lee, D.; Jang, K.; Choo, H.; Kim, T. H.; Jung, J. U.; Min, S. J.; Yang, C. S., Identification of highly potent and selective inhibitor, TIPTP, of the p22phox-Rubicon axis as a therapeutic agent for rheumatoid arthritis. *Sci Rep* **2020,** *10* (1), 4570.
